# Multifactorial Role of Mitochondria in Echinocandin Tolerance Revealed by Transcriptome Analysis of Drug-Tolerant Cells

**DOI:** 10.1128/mBio.01959-21

**Published:** 2021-08-10

**Authors:** Rocio Garcia-Rubio, Cristina Jimenez-Ortigosa, Lucius DeGregorio, Christopher Quinteros, Erika Shor, David S. Perlin

**Affiliations:** a Center for Discovery and Innovation, Hackensack Meridian Health, Nutley, New Jersey, USA; b Department of Medical Sciences, Hackensack Meridian Health School of Medicine, Nutley, New Jersey, USA; c Lombardi Comprehensive Cancer Center, Department of Microbiology and Immunology, Georgetown University, Washington, DC, USA; Duke University

**Keywords:** *Candida glabrata*, echinocandins, transcriptomics, mitochondria, antifungal drug tolerance

## Abstract

Fungal infections cause significant mortality and morbidity worldwide, and the limited existing antifungal reservoir is further weakened by the emergence of strains resistant to echinocandins, a first line of antifungal therapy. Candida glabrata is an opportunistic fungal pathogen that rapidly develops mutations in the echinocandin drug target β-1,3-glucan synthase (GS), which are associated with drug resistance and clinical failure. Although echinocandins are considered fungicidal in *Candida* sp., a subset of C. glabrata cells survive echinocandin exposure, forming a drug-tolerant cell reservoir, from which resistant mutations are thought to emerge. Despite their importance, the physiology of rare drug-tolerant cells is poorly understood. We used fluorescence-activated cell sorting to enrich for echinocandin-tolerant cells, followed by modified single-cell RNA sequencing to examine their transcriptional landscape. This analysis identified a transcriptional signature distinct from the stereotypical yeast environmental stress response and characterized by upregulation of pathways involved in chromosome structure and DNA topology and downregulation of oxidative stress responses, of which the latter was observed despite increased levels of reactive oxygen species. Further analyses implicated mitochondria in echinocandin tolerance, wherein inhibitors of mitochondrial complexes I and IV reduced echinocandin-mediated cell killing, but mutants lacking various mitochondrial components all showed an echinocandin hypotolerant phenotype. Finally, GS enzyme complexes purified from mitochondrial mutants exhibited normal *in vitro* inhibition kinetics, indicating that mitochondrial defects influence cell survival downstream of the drug-target interaction. Together, these results provide new insights into the C. glabrata response to echinocandins and reveal a multifactorial role of mitochondria in echinocandin tolerance.

## INTRODUCTION

Invasive candidiasis is an emerging life-threatening infection recognized as a major cause of morbidity and mortality (1). For decades, antifungal drugs of the azole class have been used as the primary therapy to treat *Candida* infections. Azoles have excellent activity against the predominant *Candida* species Candida albicans, and azole resistance in this species remains relatively low. However, an epidemiological shift has been taking place worldwide toward non-C. albicans species that are inherently resistant or readily acquire azole resistance, most notably Candida glabrata (2). This shift has led to the widespread use of echinocandin drugs as a first-line antifungal therapy to treat and prevent invasive candidiasis (3). Alarmingly, an increase in echinocandin-resistant C. glabrata isolates associated with clinical failure has been reported worldwide (4–7). However, the mechanisms enabling C. glabrata to develop echinocandin resistance are still very poorly understood.

Although echinocandins are fungicidal drugs in yeast, a small subset of C. glabrata cells within a given population demonstrate drug tolerance by surviving prolonged exposures to echinocandins (8, 9). Tolerance to fungicidal drugs is defined as the ability of cells to survive a drug concentration that is expected to kill cells, usually at or above the MIC (8), whereas resistance is manifested as the ability of the cells to grow in the presence of drug and is genetically stable. Clinical resistance to echinocandins is virtually always due to mutations in the hot spot regions of *FKS1* and *FKS2* genes (10). These genetic mutations leading to stable drug resistance are thought to emerge from the drug-tolerant cellular reservoir, making tolerance a key prerequisite to echinocandin resistance (8, 11). However, the molecular underpinnings of tolerance are currently not understood. Because echinocandins target β-1,3-glucan synthase (GS), an enzyme that helps build the fungal cell wall, thus far, studies of echinocandin tolerance have focused on the role of cell wall integrity pathways (12–14). A more unbiased examination of echinocandin tolerance can be derived from exploring the transcriptional landscape of echinocandin-tolerant cells. However, this approach has not been attempted thus far due to the difficulty of studying a very small subset of cells. Instead, the few studies that have examined the transcriptional response to echinocandins have used bulk-RNA isolated from entire echinocandin-treated cultures (15, 16), which are largely comprised of dead and dying cells due to the drug’s quick fungicidal action (17), thus potentially obscuring the transcriptional state of drug-tolerant cells.

In this study, we used fluorescence-activated cell sorting (FACS) to highly enrich for C. glabrata cells that have survived prolonged echinocandin exposure *in vitro*, followed by their transcriptional analysis using a modified single-cell RNA sequencing (RNA-seq) approach wherein transcriptomes of groups of several surviving cells were sequenced. This analysis identified several unique features of the transcriptional landscape of echinocandin-tolerant cells, including a downregulation of the oxidative stress response pathway, which occurs despite an echinocandin-induced increase in reactive oxygen species (ROS) abundance. We also found that, although inhibitors of mitochondrial complexes I and IV reduced both echinocandin-induced ROS formation and echinocandin-mediated cell killing, an ROS scavenger did not alter cell killing dynamics, indicating that ROS *per se* do not significantly contribute to cell death upon echinocandin exposure. Moreover, we found that mutants lacking various mitochondrial components, including both respiration-proficient and respiration-deficient strains, all showed an echinocandin hypotolerant phenotype at sub-MICs. Finally, we partially purified the echinocandin target enzyme β-1,3-glucan synthase from mitochondrial mutants and found that the enzyme’s sensitivity to echinocandins was not altered, indicating that mitochondrial status influences cell survival downstream of the echinocandin-enzyme interaction. Together, these results provide new insights into the C. glabrata response to echinocandins and reveal the involvement of mitochondria in echinocandin tolerance.

## RESULTS

### Using fluorescence-activated cell sorting to enrich for echinocandin-tolerant C. glabrata cells for RNA sequencing (RNA-seq).

**FIG 1:**
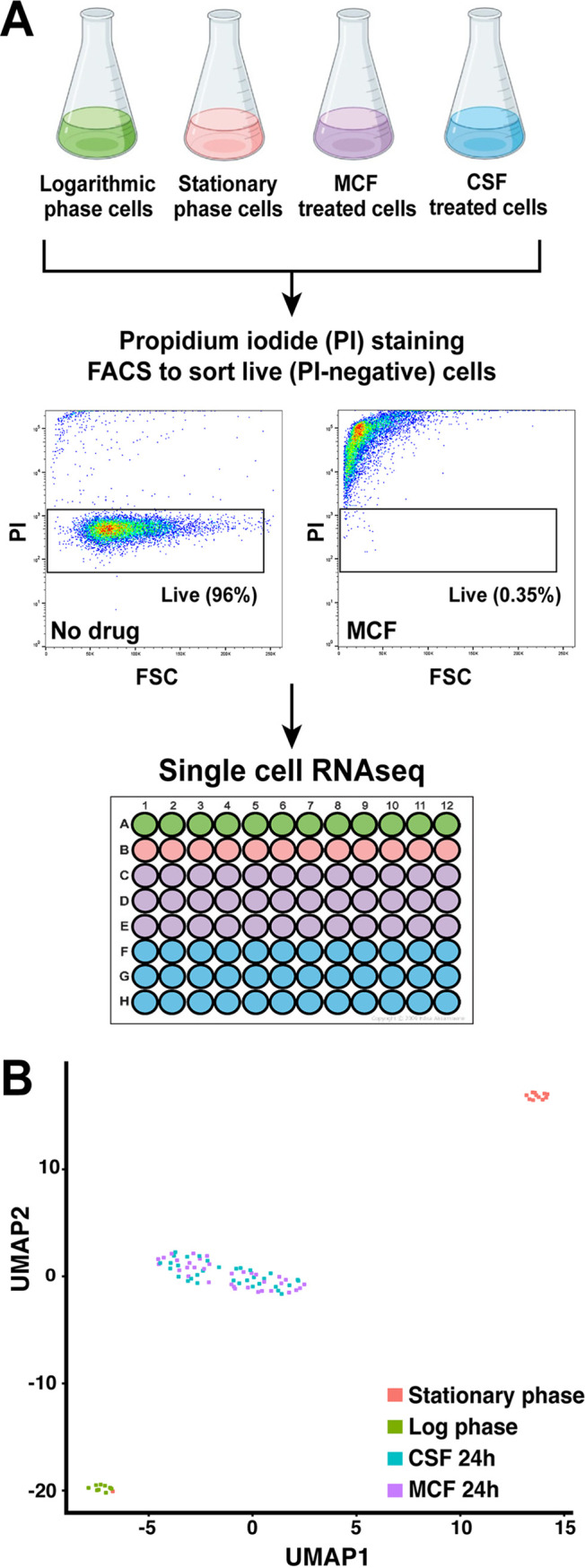
Isolation of rare echinocandin-tolerant C. glabrata cells after micafungin or caspofungin exposure followed by single-cell RNA sequencing. (A) C. glabrata cells were exposed to above-MICs of caspofungin (0.25 μg/ml) and micafungin (0.06 μg/ml) for 24 hours. Propidium iodide (PI)-negative cells, which accounted for <1% of all cells, were defined as echinocandin tolerant. Two kinds of “no-drug” controls were included, as follows: cells in either logarithmic or stationary phase. Sorted cells were analyzed using a modified single-cell RNA sequencing approach where each well contained 5 to 10 viable yeast cells. (B) Transcriptional profiles of caspofungin-tolerant cells and micafungin-tolerant cells were highly similar to each other but distinct from either stationary or logarithmic controls.

To isolate ultrarare echinocandin-tolerant C. glabrata cells for subsequent transcriptome analysis, we used fluorescence-activated cell sorting (FACS). We tested several combinations of live/dead dyes and fluorescent markers (see Table S1 in the supplemental material) in cells that had been cultured in the presence of either 0.25 μg/ml caspofungin or 0.06 μg/ml micafungin (corresponding to 2× MIC for strain ATCC 2001) for 24 hours, followed by plating of the sorted fractions on drug-free medium and counting the CFU. We found that sorting cells that did not stain with propidium iodide (PI) (Fig. 1A) resulted in the strongest enrichment for cells capable of producing colonies, from less than 1 in 1,000 events to 1 in 20 to 25 events. Thus, the PI-negative cell subset, which contained <1% of all cells, was designated containing echinocandin-tolerant cells. Next, the sorted cells were analyzed using a modified single-cell RNA sequencing approach as follows. Based on pilot experiments comprised of FACS followed by plating and CFU counts, we calculated the number of total events that had to be sorted to obtain approximately 10 viable C. glabrata cells per well (in order to ensure a robust level of RNA for sequencing). This process was done both for echinocandin-treated cell samples as well as for untreated controls. Thus, for untreated cells, the wells would contain the same number of viable cells as for echinocandin-treated cells. Although for the echinocandin-treated cells, the wells would also contain some cell debris and inviable cells, viable cells would be significantly “enriched” in these wells relative to “bulk” RNA-seq (see below). Because the echinocandin-tolerant cells were on the one hand expected to be nongrowing but on the other hand present in a rich nutritional environment, we used two kinds of “no-drug” controls, namely, cells in either logarithmic (mimicking the nutrient-rich environment) or stationary phase (mimicking the nongrowing state), stained with PI and sorted in the same fashion (Fig. 1A).

10.1128/mBio.01959-21.6 TABLE S1 Sorting cells that did not stain with propidium iodide resulted in the strongest enrichment for cells capable of producing colonies. Fluorescence-activated cell sorting (FACS) was used with different combinations of live/dead dyes and fluorescent markers in cells cultured in the presence of either 0.25 μg/ml caspofungin or 0.06 μg/ml micafungin for 24 h, followed by plating of the sorted fractions on drug-free medium and counting the colony forming units (CFUs). PI, propidium iodide; CFDA-AM, 5-carboxyfluorescein diacetate, acetoxymethyl ester; SG, Sytox green; RFP, red fluorescence protein; GFP, green fluorescence protein. Download Table S1, DOCX file, 0.01 MB. Copyright © 2021 Garcia-Rubio et al. 2021 Garcia-Rubio et al. https://creativecommons.org/licenses/by/4.0/ This content is distributed under the terms of the Creative Commons Attribution 4.0 International license.

In addition to performing RNA-seq on samples enriched for tolerant cells, we also performed bulk RNA-seq on unsorted echinocandin-treated cells as follows. A total of 0.06 μg/ml of micafungin was added to 10^7^ C. glabrata cells/ml, which were then cultured for 24 h, and their RNA was isolated and analyzed by RNA-seq. Cells grown for 24 h in the absence of micafungin (i.e., stationary-phase cells) were used as the control.

### Tolerant cell-enriched RNA-seq identified a unique transcriptional signature of echinocandin-tolerant cells.

The sorted PI-negative cells were briefly treated with zymolyase to digest the fungal cell walls, followed by analysis using the single-cell RNA-seq pipeline at the Columbia University Genome Center. As is evident from the resulting UMAP schematic, the transcriptional profiles of caspofungin-tolerant cells and micafungin-tolerant cells were highly similar to each other but distinct from either stationary or logarithmic controls (Fig. 1B). Therefore, for all the following analyses, transcriptional data from caspofungin- and micafungin-tolerant cells were grouped. Next, raw tolerant cell-enriched RNA-seq data were normalized using the sctransform package, and Seurat was used to generate a data set of individual gene expression changes in echinocandin-tolerant cells relative to either stationary or logarithmic control cells (see Data Set S1 in the supplemental material). We defined differentially expressed genes (DEGs) as showing at least one log_2_ unit (2-fold) expression difference in echinocandin-tolerant cells relative to no-drug controls. DEGs were also defined in the bulk RNA-seq data set using the same criteria.

10.1128/mBio.01959-21.10 DATA SET S1 “Bulk” and “enriched” RNA-seq data. Download Data Set S1, XLSX file, 2.5 MB. Copyright © 2021 Garcia-Rubio et al. 2021 Garcia-Rubio et al. https://creativecommons.org/licenses/by/4.0/ This content is distributed under the terms of the Creative Commons Attribution 4.0 International license.

**FIG 2:**
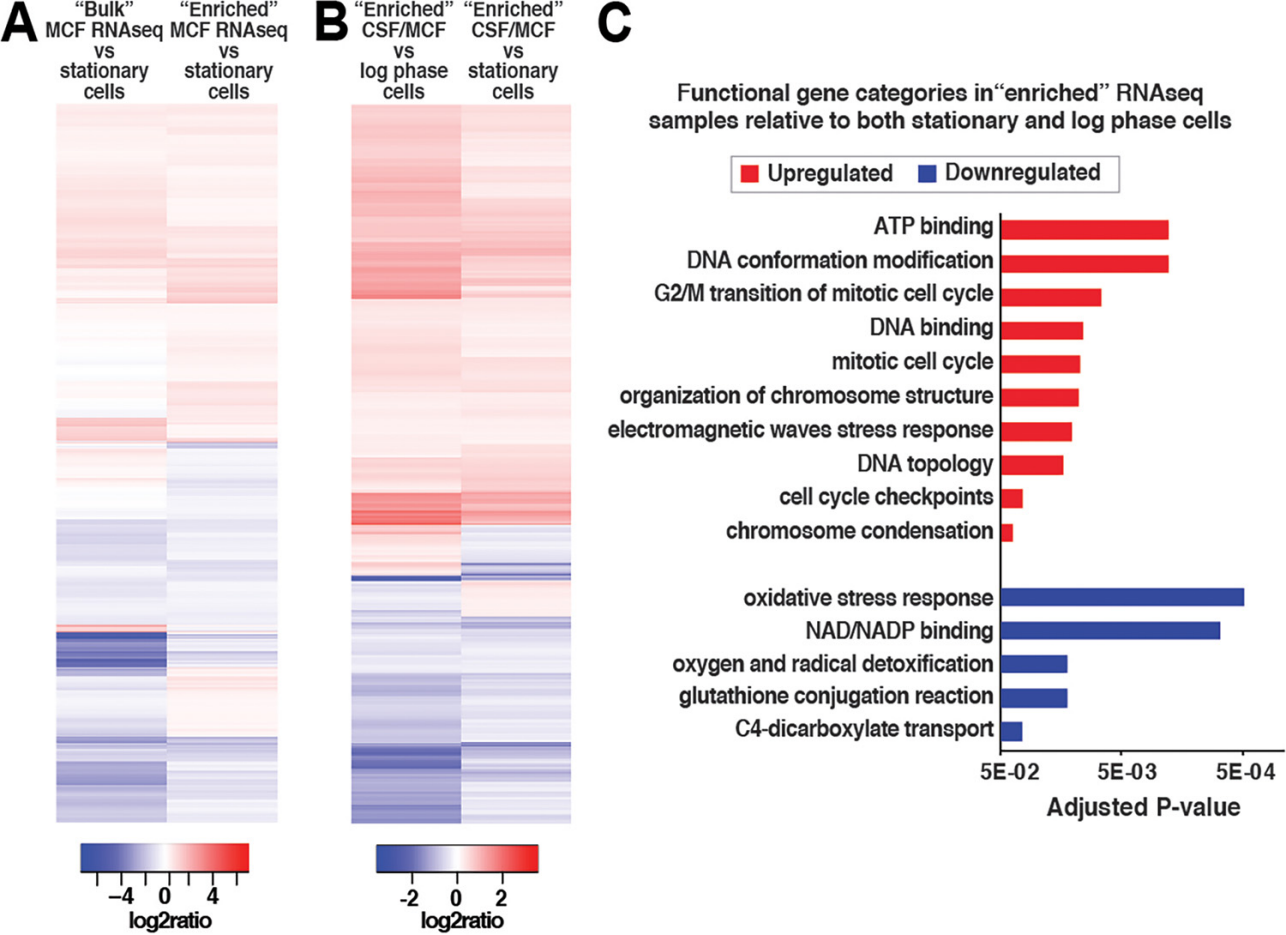
Echinocandin-tolerant C. glabrata cells upregulate genes involved in DNA topology and chromosome structure and downregulate genes involved in oxidative stress responses. (A) Heatmap showing the log_2_ ratios of bulk versus tolerant cell-enriched (enriched) RNA-seq samples. (B) Heatmap showing the log_2_ ratios of enriched RNA-seq samples relative to no-drug log-phase or stationary-phase controls. (C) Genes upregulated relative to both no-drug controls were enriched for functional categories of ATP binding, DNA conformation modification and topology, and chromosome organization, whereas downregulated genes were enriched for functional categories of oxidative stress response, NAD/NADH binding, and oxygen and radical detoxification.

A comparison of bulk and enriched RNA-seq data sets revealed a number of similarities and differences (Fig. 2A, see Fig. S1A, B in the supplemental material). Functional enrichment analysis (FUNCAT) showed that downregulated genes in all three data sets (bulk relative to stationary-phase controls, enriched relative to stationary controls, and enriched relative to log-phase controls) belonged to multiple functional categories involved in energy production and metabolism, particularly those related to mitochondrial functions (e.g., electron transport, respiration, and oxidative stress response) (Fig. S1A to C). In contrast, there was no overlap between the FUNCAT categories of upregulated genes in bulk versus enriched RNA-seq data sets (Fig. S1A versus Fig. S1B, C). The bulk RNA-seq data set contained only a few upregulated FUNCAT categories, of which all were related to cell wall metabolism (Fig. S1A), consistent with the role of echinocandins in inhibiting cell wall biogenesis. In contrast, both enriched RNA-seq data sets contained a larger number of upregulated FUNCAT gene categories, including ATP binding, DNA binding, DNA repair, DNA topology, and modification of chromosome structure (Fig. S1B to C). Interestingly, although several individual cell wall maintenance genes were upregulated in the enriched data sets (see below), the cell wall maintenance FUNCAT categories were not identified in these data sets, suggesting that enriching for surviving cells does not necessarily enrich for cells with transcriptionally upregulated cell wall integrity pathways.

10.1128/mBio.01959-21.1 FIG S1 Functional categories enriched among differentially upregulated and downregulated genes. (A) Functional categories in “bulk” RNA-seq samples relative to stationary “no-drug” control. (B) Functional categories in tolerant cell-enriched (“enriched”) RNA-seq samples relative to stationary no-drug controls. (C) Functional categories in tolerant cell-enriched (enriched) RNA-seq samples relative to log phase no-drug controls. Functional categories found in at least two datasets are marked in violet. Download FIG S1, TIF file, 2.0 MB. Copyright © 2021 Garcia-Rubio et al. 2021 Garcia-Rubio et al. https://creativecommons.org/licenses/by/4.0/ This content is distributed under the terms of the Creative Commons Attribution 4.0 International license.

For the following analyses, we focused on the RNA-seq data obtained from samples enriched for echinocandin-tolerant cells. Comparisons of echinocandin-tolerant cells to either log-phase or stationary-phase controls resulted in similar but not identical expression patterns and FUNCAT categories (Fig. 2B, Fig. S1B and C), as is also evident from UMAP analysis (Fig. 1B). We were particularly interested in the genes that were upregulated or downregulated relative to both no-drug controls (Data Set S1). The upregulated gene set was enriched for functional categories of ATP binding, DNA conformation modification and topology, and chromosome organization (Fig. 2C). Consistent with echinocandins targeting β-1,3-glucan synthase (GS), an enzyme essential for maintaining cell wall structure, several genes involved in cell wall biosynthesis were upregulated in echinocandin-tolerant cells, including *FKS1* and *FKS2*, which encode GS, as well as *KRE5*, which encodes a protein required for β-1,6 glucan biosynthesis. Interestingly, several genes encoding multidrug ABC transporters (*CDR1* and *YOR1*) were also among the upregulated group, even though multidrug transporters are not thought to mediate resistance to echinocandins (18, 19). Genes downregulated relative to both no-drug controls were enriched for functional categories of oxidative stress response, NAD/NADH binding, and oxygen and radical detoxification (Fig. 2C). Of note, this finding is distinct from the stereotypical yeast environmental stress response, where these functional gene categories are typically upregulated (20, 21), underscoring that the transcriptional status of echinocandin-tolerant cells is unique and distinct from other types of stressed cells.

### Mitochondrial inhibitors but not topoisomerase inhibitors increase echinocandin tolerance in C. glabrata.

**FIG 3:**
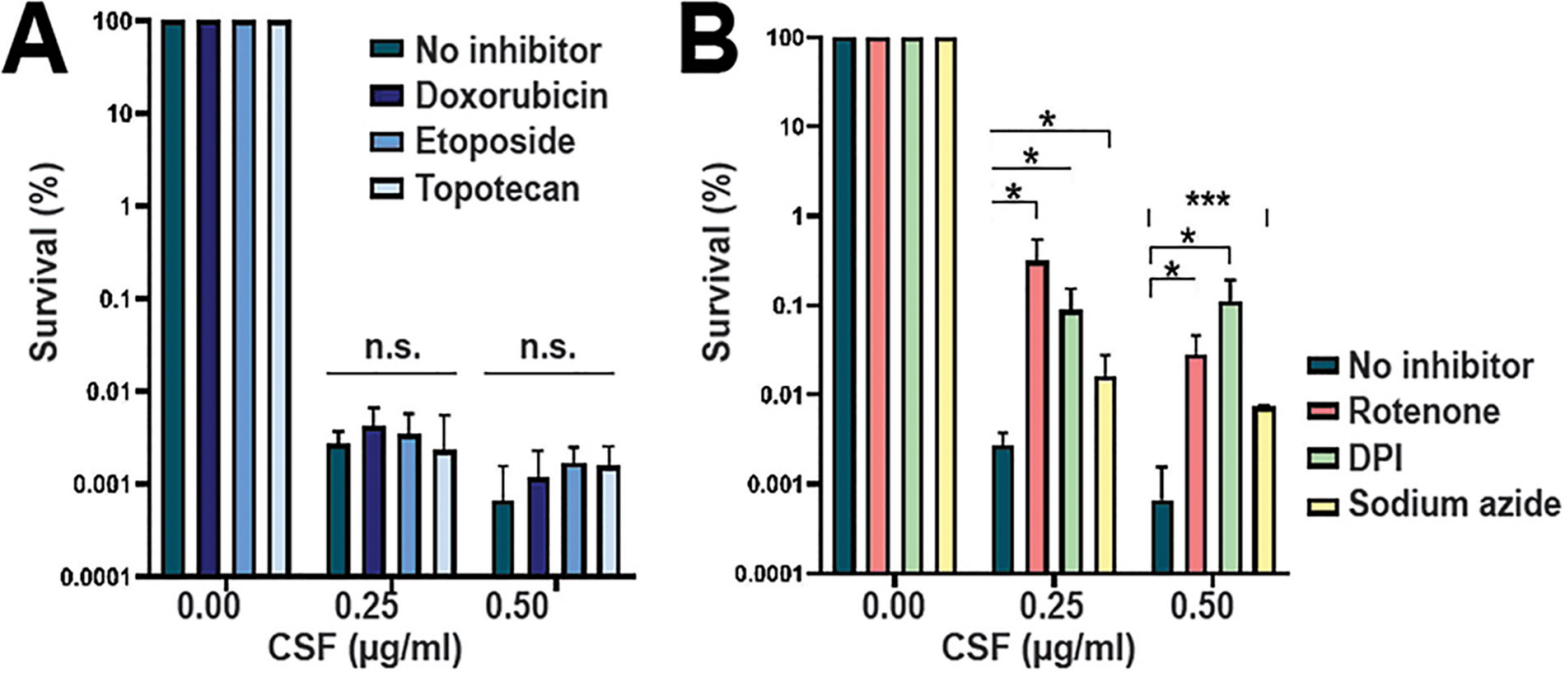
Mitochondrial inhibitors but not topoisomerase inhibitors increase echinocandin tolerance in C. glabrata. Chemical inhibitors were used to modulate the most significantly up- or downregulated pathways and examine the resulting effects on echinocandin tolerance. Topoisomerase inhibitors did not alter echinocandin tolerance in C. glabrata (A), whereas mitochondrial inhibitors increased the fraction of caspofungin-surviving cells (B). Statistical significance was calculated using an unpaired *t* test and is indicated with asterisks (*, *P* ≤0.05; **, *P* ≤ 0.01; ***, *P* ≤ 0.001; n.s., not significant).

Based on the functional categories of upregulated and downregulated genes, we selected different types of chemical inhibitors to modulate the most significantly up- or downregulated pathways and ask whether this modulation altered C. glabrata echinocandin tolerance. We note that because caspofungin- and micafungin-tolerant cells had similar transcriptional profiles (Fig. 1B), the experiments described below, especially the most laborious ones, were performed using only caspofungin. First, we treated C. glabrata with inhibitors of topoisomerase I (topotecan) or II (doxorubicin and etoposide) together with caspofungin for 24 hours. Etoposide and doxorubicin were used at concentrations corresponding to one-third and one-quarter of their MIC in C. glabrata, respectively. Topotecan has been described to have no antifungal effect and to not affect viability, so we used 100 μM as did previous studies (22, 23) (see Table S2 in the supplemental material). We found that the addition of these topoisomerase inhibitors to cells cultured in the presence of caspofungin did not alter the C. glabrata tolerance phenotype (Fig. 3A).

10.1128/mBio.01959-21.7 TABLE S2 MIC susceptibility testing of a selected concentration of inhibitors in combination with caspofungin and micafungin showed identical MIC values to the two echinocandins themselves (i.e., caspofungin and micafungin, respectively). The inhibitor concentration selected was based on the MIC value obtained in the susceptibility testing together with its solubility in YPD liquid media. DPI, diphenyleneiodonium chloride. *Rotenone is nonsoluble at >0.3 mM. **Other authors have used 2.5 to 10 mM of ascorbic acid combined with other drug compounds in *Candida* species (C. F. Rodrigues and M. Henriques, Ther Adv Infect Dis 4(1):10–17, 2017, doi:https://doi.org/10.1177/2049936116684477; F. Van Hauwenhuyse, A. Fiori, and P. Van Dijck, Eukaryot Cell 13:1278–1289, 2014, doi:https://doi.org/10.1128/EC.00096-14; D. Vandenbosch, K. Braeckmans, H. J. Nelis, and T. Coenye, J Antimicrob Chemother 65(4):694–700, 2010, doi:https://doi.org/10.1093/jac/dkq019). Download Table S2, DOCX file, 0.01 MB. Copyright © 2021 Garcia-Rubio et al. 2021 Garcia-Rubio et al. https://creativecommons.org/licenses/by/4.0/ This content is distributed under the terms of the Creative Commons Attribution 4.0 International license.

Next, we asked whether inhibitors of mitochondrial enzymes that alter reactive oxygen species (ROS) production would alter tolerance to caspofungin. We used inhibitors of mitochondrial complex I (rotenone and diphenyleneiodonium chloride [DPI]) and complex IV (sodium azide) at concentrations corresponding to one-third of their MIC in C. glabrata for DPI and sodium azide. The rotenone concentration used (0.3 mM) was determined by the highest concentration at which it was soluble in yeast extract-peptone-dextrose (YPD) medium (Table S2). We observed that these inhibitors increased the fraction of cells surviving after 24 hours of treatment by 10- to 100-fold (Fig. 3B). Both complex I inhibitors (rotenone and DPI) improved survival more than the complex IV inhibitor sodium azide.

### Caspofungin treatment leads to an increase in reactive oxygen species production but a downregulation of the oxidative stress response.

**FIG 4:**
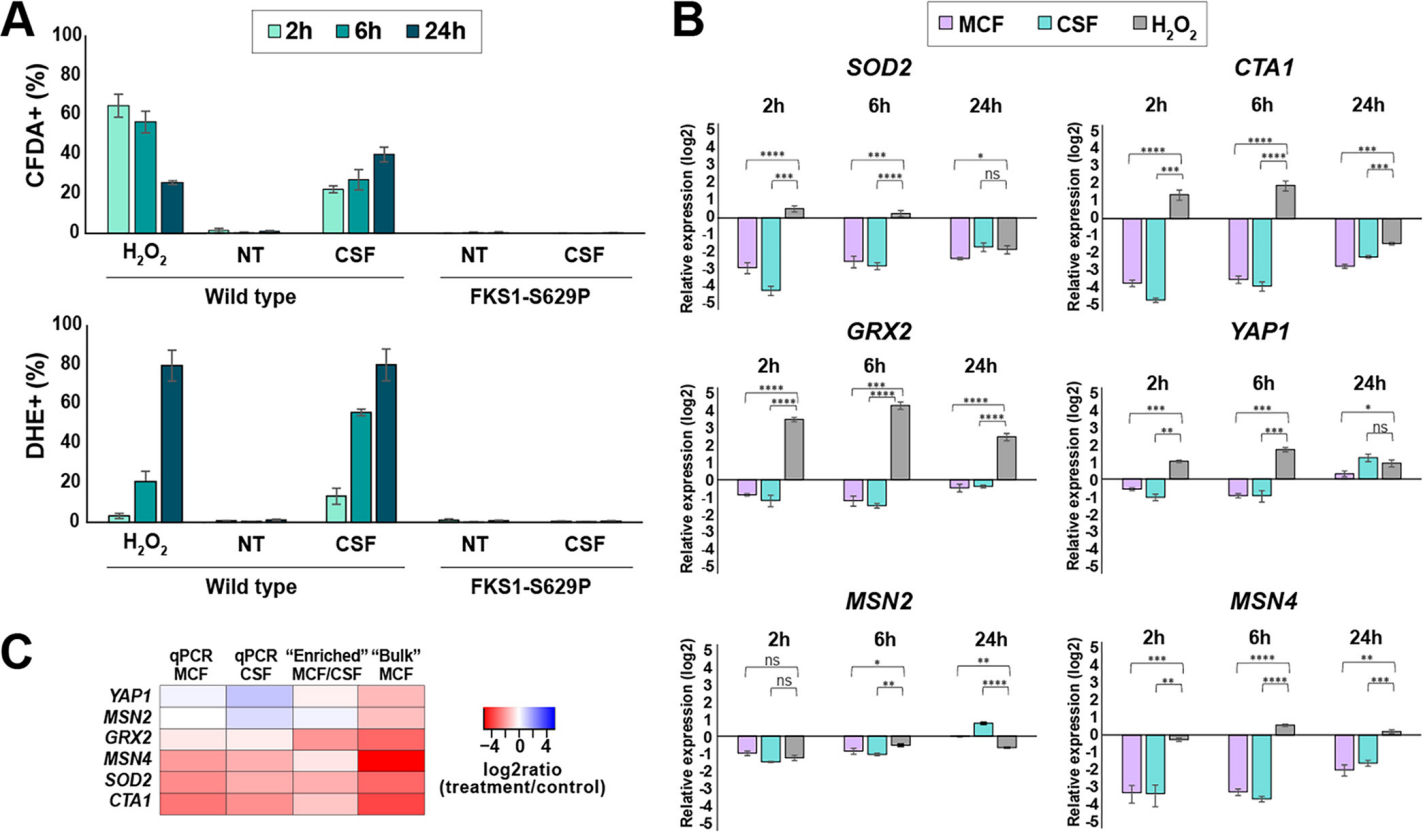
Caspofungin treatment leads to increased reactive oxygen species (ROS) levels but decreased expression of genes involved in oxidative stress responses. (A) Two different ROS-sensitive dyes, namely, CFDA or DHE, were used to detect hydrogen peroxide or superoxide species, respectively. Dead cells were gated out using PI or Sytox green (SG). A total of 50 mM H_2_O_2_ was used as a positive control. Caspofungin induced a strong increase in both CFDA and DHE staining, and this increase was abolished in an *fks1* mutant, which lacks wild-type glucan synthase (echinocandin target enzyme). NT, caspofungin nontreated cells. (B) Oxidative stress response (OSR) genes as well as transcription factors involved in the regulation of the OSR pathway were downregulated by caspofungin and micafungin treatment but were either induced or unaltered by the H_2_O_2_ control. (C) Heatmap comparing log_2_ ratios of treatment/control obtained using different molecular approaches for the six selected genes involved in oxidative stress responses. The statistical significance was calculated using an unpaired *t* test and is indicated with asterisks (*, *P* ≤ 0.05; **, *P* ≤ 0.01; ***, *P* ≤ 0.001; ****, *P* ≤ 0.0001; ns, not significant).

Increased ROS have been observed as a consequence of drug treatment both in fungi and bacteria and have been proposed as a key mediator of drug-induced killing in some of these systems (24–26). This proposition, together with the observation that mitochondrial inhibitors improve echinocandin tolerance, led us to examine ROS levels and ROS responses in caspofungin-treated cells more closely. A typical method to detect ROS is by using ROS-sensitive dyes followed by flow cytometry analysis. We used two different dyes, namely, 2′,7′-dichlorodihydrofluorescein diacetate (CFDA) and dihydroethidium (DHE), which are thought to detect the specific ROS hydrogen peroxide and superoxide, respectively (27). However, the exact nature of the species detected by these dyes, especially CFDA, is still obscure (28). In these experiments, C. glabrata cells were treated with the indicated agents for 2, 6, or 24 hours, followed by staining with PI or Sytox green to gate out dead cells and CFDA or DHE to detect ROS (PI was used with CFDA and Sytox green was used with DHE due to color compatibility). We used treatment with 50 mM H_2_O_2_ as a positive control and an echinocandin-resistant *fks1* mutant as a negative control. As expected, we detected a strong increase in both CFDA and DHE staining induced by H_2_O_2_ treatment, albeit with different dynamics; CFDA staining peaked at 2 hours of exposure and then declined over 24 hours, whereas DHE staining gradually increased over 24 hours (Fig. 4A). Importantly, we observed that 0.25 μg/ml caspofungin also induced a strong increase in both CFDA and DHE staining, with staining intensity of both dyes increasing over the 24-hour period. This increase was entirely dependent on the presence of a wild-type GS enzyme, as no increase was observed in the *fks1* mutant (Fig. 4A). Thus, we concluded that caspofungin-mediated inhibition of GS activity resulted in an increase in cellular ROS levels.

The observed increase in ROS but a decrease in the expression of genes involved in oxidative stress responses (Fig. 2C, Fig. S1) by RNA-seq was surprising, so we probed it further by examining the transcriptional levels of several individual genes during caspofungin or micafungin treatment by quantitative reverse transcriptase PCR (qRT-PCR). These experiments were performed by harvesting bulk RNA from echinocandin-treated or untreated cultured, followed by qPCR analysis. We chose genes with well-established roles in the oxidative stress response (OSR), such as mitochondrial manganese superoxide dismutase (*SOD2*, *CAGL0E04356g*), a catalase A (*CTA1*, *CAGL0K10868g*), and a glutathione oxidoreductase (*GRX2*, *CAGL0K05813g*), as well as three transcription factors that regulate the OSR pathway (*YAP1*, *CAGL0H04631g*; *MSN2*, *CAGL0F05995g*; and *MSN4*, *CAGL0M13189g*) (29). The relative expression of each gene was normalized to a control locus (*RDN5.8*) and calculated relative to that of untreated stationary-phase cells. H_2_O_2_ treatment was used as a control. Consistent with the RNA-seq results (Fig. 2C), we observed that transcript levels of all these genes were downregulated by caspofungin and micafungin treatment, with *SOD2*, *CTA1*, and *MSN4* showing the greatest reduction in RNA abundance (up to 4 log_2_ units, or 16-fold) (Fig. 4B). This transcriptional response was highly distinct from that induced by H_2_O_2_, which predominantly resulted either in an increase or no change in the transcript abundance of these genes (Fig. 4B). The log_2_ ratios (treatment/no treatment) obtained for these genes by qPCR showed good agreement with bulk and enriched RNA-seq results (Fig. 4C).

### ROS reduction *per se* does not increase echinocandin tolerance in C. glabrata.

**FIG 5:**
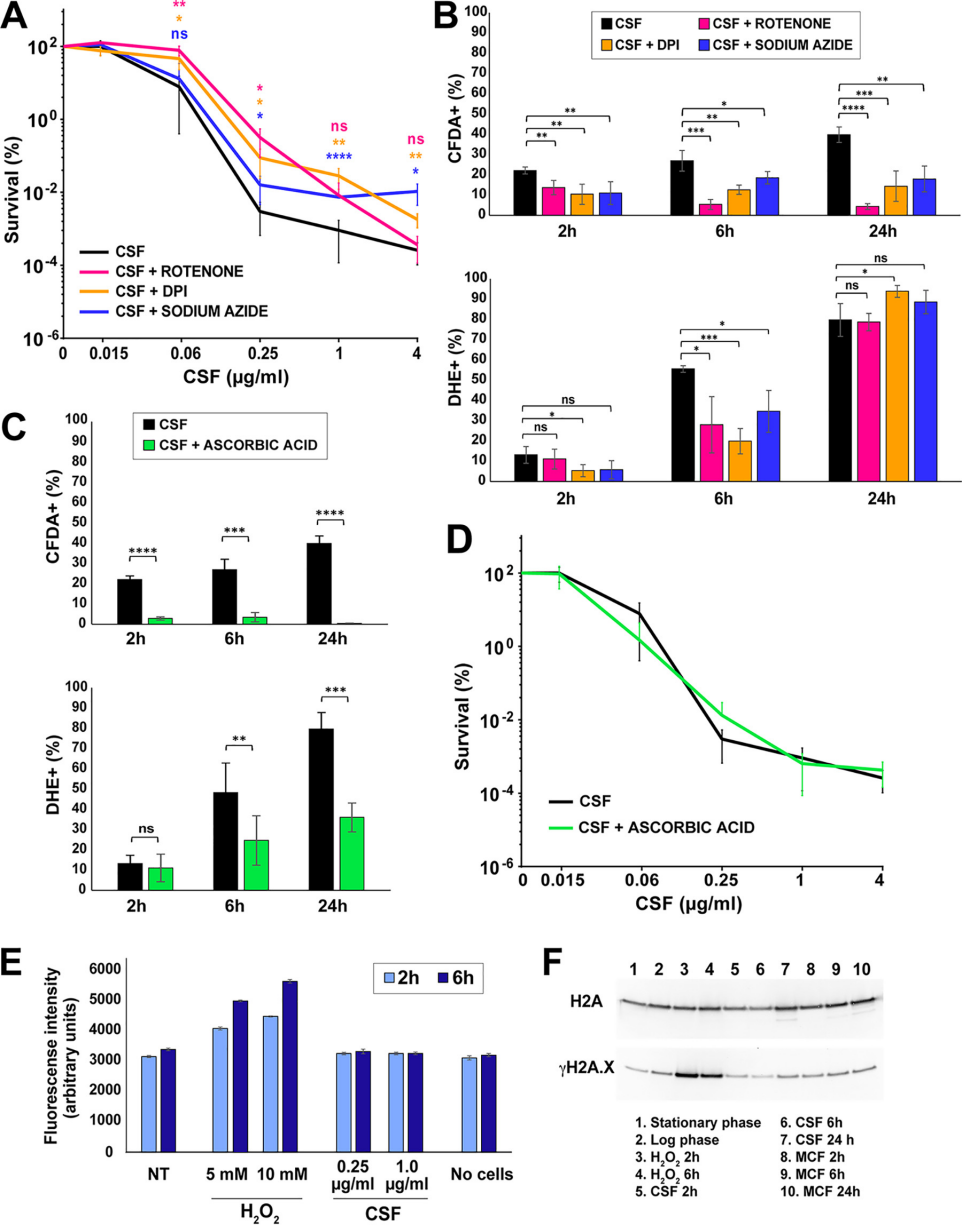
Mitochondrial inhibitors but not an ROS scavenger improve the survival of C. glabrata cells in caspofungin. (A) Mitochondrial inhibitors rotenone, DPI, and sodium azide increased the fraction of caspofungin-surviving cells over a wide range of caspofungin concentrations. (B) The mitochondrial inhibitors also reduced ROS production as detected by CFDA and DHE. (C) Ascorbic acid, an ROS scavenger, strongly decreased ROS levels induced by caspofungin. (D) Ascorbic acid did not alter cell survival in the presence of caspofungin. (E) Despite the elevation of ROS, no increase in lipid peroxidation, as measured by the DPPP fluorometric assay, was detected in the presence of caspofungin. (F) No increase in DNA damage as measured by γH2A.X abundance was detected in the presence of caspofungin or micafungin. The statistical significance was calculated using an unpaired *t* test and is indicated with asterisks (*, *P* ≤ 0.05; **, *P* ≤ 0.01; ***, *P* ≤ 0.001; ****, *P* ≤ 0.0001; ns, not significant).

As mentioned above, ROS are generally viewed as cell-damaging agents and have been shown to mediate the killing effects of certain antimicrobial drugs. To investigate whether this is the case for echinocandins in C. glabrata, we turned our focus again to the mitochondrial inhibitors. First, we examined their effects on caspofungin-induced killing of C. glabrata over a wide range of caspofungin concentrations (Fig. 5A). We believe such a killing assay is a better indicator of drug tolerance than the standard MIC determination, which measures growth but not survival in the presence of a drug (8). The caspofungin MIC value obtained for ATCC 2001 was 0.12 μg/ml, and it was not affected by any of the mitochondrial inhibitors (Table S2). Nevertheless, survival after 24 hours in caspofungin was rescued by rotenone, DPI, and sodium azide at several different caspofungin concentrations (Fig. 5A). Next, we examined how ROS production was affected by these mitochondrial inhibitors and found that they reduced ROS levels in caspofungin-treated cells (Fig. 5B). This finding was especially evident when ROS was detected by CFDA staining but less so when DHE staining was used, suggesting that these compounds reduce the abundance of some ROS more than others.

The result above was consistent with the hypothesis that elevated ROS may contribute to echinocandin-induced cell death in C. glabrata. To test this hypothesis directly, we used ROS scavengers. We tested several compounds with ROS scavenging activities (thiourea, dimethylthiourea, sodium dithionite, catalase, α-tocopherol, and ascorbic acid) but found that only ascorbic acid significantly reduced the levels of ROS in caspofungin-treated cells (Fig. 5C). Interestingly, when we measured cell survival of caspofungin treatment in the presence of ascorbic acid, we found no difference between cells cultured in the absence or presence of this ROS scavenger (Fig. 5D). This result indicated that it was not ROS *per se* that was responsible for cell death upon caspofungin treatment.

ROS can damage different cellular biomolecules, which is thought to contribute to their involvement in cell death in other contexts (30). Thus, we examined whether elevated ROS levels in caspofungin-treated cells were associated with detectable damage of two types of biomolecules, namely, lipids and DNA. To measure lipid peroxidation, a fluorometric assay was performed using diphenyl-1-pyrenylphosphine (DPPP), a compound that reacts with lipid hydroperoxides to produce fluorescent DPPP oxide. To measure DNA damage, we examined the abundance of histone H2A phosphorylated at serine 129 (γH2A.X), which is a universal marker of double-strand breaks (31). In both cases, H_2_O_2_ was used as a positive control. Interestingly, H_2_O_2_-treated cells showed a robust induction of lipid peroxidation and γH2A.X levels, whereas echinocandin-treated cells did not show evidence of either lipid peroxidation or DNA damage relative to nontreated controls (Fig. 5E and F). This result was consistent with the conclusion that despite an increased abundance of ROS in echinocandin-treated cells, these ROS did not significantly contribute to cellular damage or lethality.

### Deletion mutants lacking components of the mitochondrial respiratory chain show a caspofungin hypotolerant phenotype.

The observations that mitochondrial inhibitors altered echinocandin tolerance but that ROS *per se* were not involved made us examine the role of mitochondria in echinocandin tolerance more closely. To this end, we generated a number of different mitochondria-deficient strains (see Table S3 in the supplemental material). First, we created deletions of the following five genes encoding components of the mitochondrial respiratory chain: *NDI1* (*CAGL0B02431g*), whose Saccharomyces cerevisiae ortholog has NADH dehydrogenase activity and is a component of the fungal equivalent of mitochondrial complex I; *COX4* (*CAGL0L06160g*), whose ortholog has the cytochrome-c oxidase activity of mitochondrial complex IV; and three components of the mitochondrial complex V ATP synthase, namely, the F1 alpha subunit of the F1F0-ATPase complex encoded by *ATP1* (*CAGL0M09581g*), the F1 beta subunit encoded by *ATP2* (*CAGL0H00506g*), and the assembly factor for the F0 sector of mitochondrial F1F0 ATP synthase encoded by *ATP10* (*CAGL0C02651g*). All of these deletion mutants, with the exception of *ndi1Δ*, showed a petite phenotype characterized by slow growth and an inability to use a nonfermentable carbon source (Fig. S2). We also isolated several respiration-deficient (i.e., petite) mutants that spontaneously but rarely (approximately 1 in 3,000 to 5,000 colonies) emerged after 24 h of 0.25-μg/ml caspofungin treatment. Finally, we generated several petite mutants by treatment with ethidium bromide (32). All isolated petite mutants were respiration deficient and slow growing; however, all of them stained to various degrees by MitoTracker green, indicating that they were not fully lacking mitochondria (see Fig. S3A in the supplemental material). As expected, most mitochondrial mutants, including several gene deletions (*atp1Δ*, *atp2Δ*, and *atp10Δ*) and all petite mutants, showed elevated MICs to fluconazole (Table S3), consistent with previous reports that mitochondrial dysfunction in C. glabrata induces *PDR1* expression and promotes azole resistance (33, 34).

10.1128/mBio.01959-21.2 FIG S2 Mitochondrial defective mutants showed wild-type sensitivity to cell wall-damaging agents. Mitochondrial deletion mutants were photographed after 24 h. Petite mutants of unknown etiology, which were very slow growing, were photographed after 48 h. YPG, YP + 2% glycerol. Congo red (CR) was used at 0.05 mg/ml and calcofluor white (CW) was used at 0.01 mg/ml. Download FIG S2, TIF file, 2.4 MB. Copyright © 2021 Garcia-Rubio et al. 2021 Garcia-Rubio et al. https://creativecommons.org/licenses/by/4.0/ This content is distributed under the terms of the Creative Commons Attribution 4.0 International license.

10.1128/mBio.01959-21.3 FIG S3 Characterization of C. glabrata mitochondrial mutants. (A) MitoTracker Green detection by fluorescence microscopy showed mitochondrial content in petite C. glabrata strains. (B) Ethidium bromide-derived petite mutants were hypotolerant at sub-MIC caspofungin concentrations and showed variable survival at above-MIC caspofungin concentrations. (C) Deletion mutants lacking mitochondrial proteins not directly involved in the respiratory chain showed a caspofungin hypotolerant phenotype. The examined deletion mutants were a major ADP/ATP carrier of the mitochondrial inner membrane (*PET9*), a translocase of the inner mitochondrial membrane (*TIM18*), and a catalytic subunit of i-AAA protease complex responsible for degradation of misfolded mitochondrial gene products (*YME1*). Download FIG S3, TIF file, 1.6 MB. Copyright © 2021 Garcia-Rubio et al. 2021 Garcia-Rubio et al. https://creativecommons.org/licenses/by/4.0/ This content is distributed under the terms of the Creative Commons Attribution 4.0 International license.

10.1128/mBio.01959-21.8 TABLE S3 List of Candida glabrata strains used in this study and their fluconazole susceptibilities. ECH, echinocandins. Download Table S3, DOCX file, 0.01 MB. Copyright © 2021 Garcia-Rubio et al. 2021 Garcia-Rubio et al. https://creativecommons.org/licenses/by/4.0/ This content is distributed under the terms of the Creative Commons Attribution 4.0 International license.

**FIG 6:**
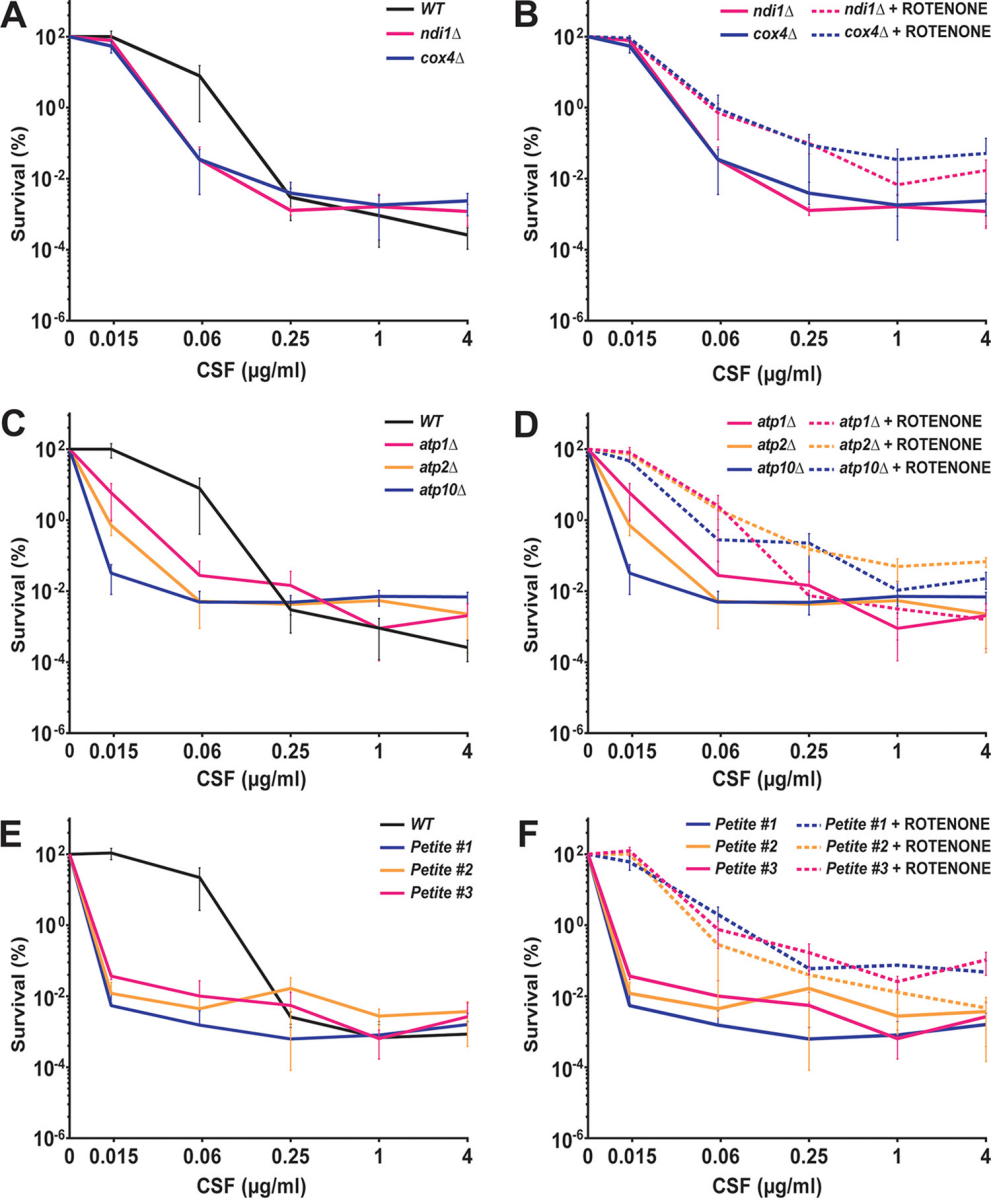
Mutants lacking various mitochondrial respiratory chain components and mitochondrial-defective petite mutants showed a caspofungin hypersusceptible phenotype, which was rescued by rotenone. Deletion mutants of mitochondrial respiratory chain component *NDI1* (NADH dehydrogenase, the yeast equivalent of complex I) and *COX4* (cytochrome-c oxidase of complex IV) showed a mild hypersusceptibility to caspofungin (A) that was rescued by rotenone (B). Deletion mutants of three components of the mitochondrial complex V ATP synthase (*ATP1*, *ATP2*, and *ATP10*) also showed a hypersusceptible phenotype to a wide range of caspofungin concentrations below the MIC value (C) that was rescued by rotenone (D). Respiration-deficient petite mutants generated during caspofungin exposure showed the strongest hypersusceptible phenotypes (E) that were also rescued by rotenone (F).

All mitochondria-deficient mutants generated above were examined in the caspofungin killing assay. Interestingly, and contrary to the expectation formed based on the phenotype of mitochondrial inhibitors, all deletion mutants (including *ndi1Δ*, which was not petite) showed a caspofungin hypotolerant phenotype at one or more concentrations (Fig. 6A and B). The same hypotolerant phenotype was observed in all examined petite mutants (Fig. 6C and Fig. S3B). Because this hypotolerance was different from the phenotype caused by inhibitors of the mitochondrial respiratory chain, we wondered whether it may be due not to a lack of respiratory genes *per se* but to general mitochondrial defects. To test this hypothesis, we also deleted the following three genes encoding mitochondrial proteins not directly involved in the respiratory chain: *PET9* (*CAGL0F04213g*), encoding the major ADP/ATP carrier of the mitochondrial inner membrane; *TIM18* (*CAGL0A03784g*), encoding a translocase of the inner mitochondrial membrane; and *YME1* (*CAGL0K05093g*), encoding a catalytic subunit of i-AAA protease complex responsible for degradation of misfolded mitochondrial gene products. We found that each of these mutants was likewise hypotolerant to caspofungin, indicating that this hypotolerance may be a general feature of C. glabrata strains with mitochondrial defects (Fig. S3C).

Interestingly, the hypotolerance of mitochondrial mutants to caspofungin was rescued by rotenone in every tested case (Fig. 6D to F), indicating that rotenone’s effect on echinocandin tolerance is exerted whether cells have functional mitochondria or not. In contrast, DPI did not improve survival in any examined mitochondrial mutants, indicating that its effect was mediated by mitochondria (see Fig. S4A, B in the supplemental material). Furthermore, we examined the effect of sodium azide on the *cox4Δ* mutant because complex IV is the target of this mitochondrial inhibitor. No rescue of survival was observed, indicating that a functional complex IV is necessary to mediate the effect of sodium azide (Fig. S4C).

10.1128/mBio.01959-21.4 FIG S4 Diphenyleneiodonium chloride (DPI) and sodium azide did not improve survival in deletion mutants lacking mitochondrial respiratory chain components. Deletion mutants of mitochondrial respiratory chain components *NDI1* (NADH dehydrogenase of equivalent of complex I) and *COX4* (cytochrome-c oxidase of complex IV) (A), and complex V ATP synthase (*ATP1*, *ATP2*, and *ATP10*) (B) were not rescued by DPI in the caspofungin tolerance assay. (C) Sodium azide did not rescue the survival of the *cox4Δ* mutant. Download FIG S4, TIF file, 1.4 MB. Copyright © 2021 Garcia-Rubio et al. 2021 Garcia-Rubio et al. https://creativecommons.org/licenses/by/4.0/ This content is distributed under the terms of the Creative Commons Attribution 4.0 International license.

To test whether the hypotolerance to caspofungin was due to a general cell wall integrity defect, we assayed the sensitivity of mitochondrial mutants to two different cell wall-damaging agents, namely, Congo red and calcofluor white. We found that none of the examined mutants were more sensitive to these agents than the parental wild-type strains (Fig. S2), indicating that the hypotolerance effect was specific to echinocandin-mediated cell killing.

Because the effect of mitochondrial inhibitors (which increased tolerance) was opposite of that of mitochondrial mutants (which reduced tolerance), we asked whether one important difference between the two was the timing of the onset of mitochondrial dysfunction. In the experiments using mitochondrial inhibitors, the inhibitors had been added to cells with intact mitochondria at the same time as the echinocandins. However, in the mutants, the mitochondria had been compromised by mutation prior to addition of echinocandins. Therefore, we asked whether inhibition of mitochondria prior to echinocandin exposure would elicit different effects relative to mitochondrial inhibition induced at the same time as echinocandin exposure. We cultured C. glabrata for 18 h in the presence of rotenone or DPI, and then we collected the cells and cultured them in fresh YPD media with the mitochondrial inhibitor and caspofungin for another 24 h, as in the standard tolerance assay (thus, rotenone or DPI was present in the culture both before and after caspofungin addition). Interestingly, pre-exposure of C. glabrata to DPI abolished the rescuing effect of DPI on caspofungin tolerance at all tested concentrations, and this finding was also true for rotenone at one sub-MIC caspofungin concentration (0.06 μg/ml) (see Fig. S5 in the supplemental material). These results suggest that chronic mitochondrial inhibition or dysfunction reduces C. glabrata echinocandin tolerance, whereas acute mitochondrial inhibition may enhance tolerance.

10.1128/mBio.01959-21.5 FIG S5 Exposure of C. glabrata cells to the mitochondrial inhibitors rotenone (A) and diphenyleneiodonium chloride (DPI) (B) prior to caspofungin treatment reduced cell survival. Rotenone was still able to rescue growth at several caspofungin concentrations above the MIC. Download FIG S5, TIF file, 0.9 MB. Copyright © 2021 Garcia-Rubio et al. 2021 Garcia-Rubio et al. https://creativecommons.org/licenses/by/4.0/ This content is distributed under the terms of the Creative Commons Attribution 4.0 International license.

### Glucan synthase enzyme from mitochondrial mutants exhibits normal echinocandin sensitivity *in vitro*.

**FIG 7:**
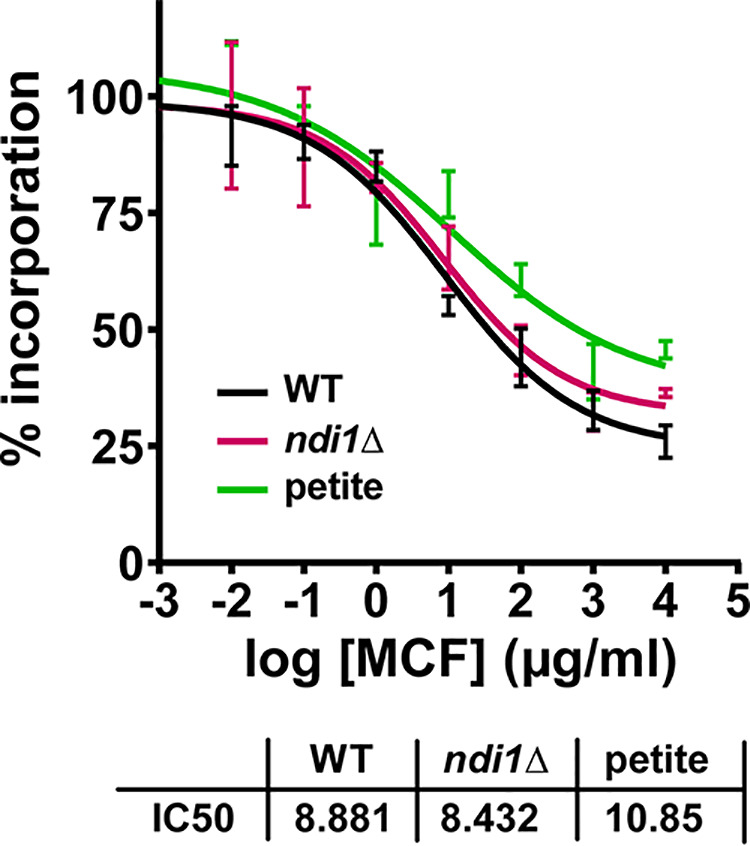
1,3-β-d-Glucan synthase (GS) isolated from mitochondrial mutants shows unaltered micafungin inhibition kinetics *in vitro*. Echinocandin inhibition profiles and biochemical sensitivity (IC_50_) for product-entrapped GS enzyme complexes were assessed by the incorporation of [^3^H]glucose into the radiolabeled product. Micafungin titration curves and 50% effective concentration (EC_50_) values are shown for two different mitochondrial mutants—the *ndi1Δ* strain and one petite mutant—and the wild-type control strain.

Echinocandins kill fungal cells by targeting the enzyme 1,3-β-d-glucan synthase (GS) and inhibiting fungal cell wall formation. Thus, the increased echinocandin susceptibility of mitochondrial mutants could potentially be due to changes in GS, which would make the enzyme less susceptible to drug action, as we had demonstrated with echinocandin resistance in Aspergillus fumigatus (35), or to changes in pathways acting downstream of GS inhibition. To distinguish between these possibilities, we partially purified GS complexes using our established method consisting of membrane protein extraction and partial GS purification by product entrapment (36) from two different mitochondrial mutants—the *ndi1Δ* strain and one petite mutant—and a wild-type control strain. The activity of the isolated GS complexes and their inhibition by micafungin were assayed *in vitro* using a β-glucan polymerization assay (36). We observed that GS isolated from mitochondrial mutants had very similar inhibition kinetics to GS isolated from the wild-type strain (Fig. 7), indicating that the effect of mitochondria on echinocandin sensitivity likely occurs downstream of the GS inhibition step.

## DISCUSSION

**FIG 8:**
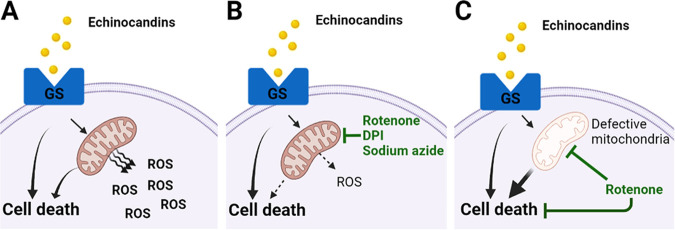
Graphical representation of the proposed model for the role of mitochondria in echinocandin tolerance. (A) Echinocandin exposure in Candida glabrata cells inhibits GS activity, leading to cell death via mitochondria-dependent as well as mitochondria-independent mechanisms. The mitochondrial involvement also results in increased ROS production. (B) When echinocandin exposure is combined with mitochondrial inhibitors, mitochondria-dependent mechanisms of cell death are reduced, along with ROS production. (C) Chronic mitochondrial dysfunction leads to hypotolerance to echinocandins characterized by decreased survival at below-MICs. In this case, rotenone still partially rescues survival, likely due to nonmitochondrial mechanisms. Created with BioRender.com.

Our study presents the first examination of the transcriptional landscape of the echinocandin-tolerant cell subset in the human pathogen C. glabrata, which is presumed to serve as a precursor for stable drug resistance (8, 11). We show that the transcriptional state of cells that have survived 24 h of above-MIC echinocandin exposure is distinct from the stereotypical transcriptional response to environmental stresses. In particular, echinocandin-tolerant cells had upregulated pathways involved in chromosome structure and DNA topology and downregulated pathways involved in oxidative stress response. This downregulation occurred even though C. glabrata cells had significantly elevated ROS levels upon echinocandin exposure. Interestingly, the ROS did not appear to contribute to echinocandin-induced cell death, as reducing ROS using ascorbic acid did not alter cell killing. Finally, we identified a multifactorial role of mitochondria in C. glabrata echinocandin tolerance; using inhibitors of respiratory chain components increased echinocandin tolerance, whereas deletion of respiratory chain components made C. glabrata cells hypotolerant to echinocandins. Together, these results provide new insights into the C. glabrata response to echinocandins and reveal the involvement of mitochondria in echinocandin tolerance. A model describing the proposed role of mitochondria in echinocandin-induced cell death is shown in Fig. 8.

Several gene categories upregulated in echinocandin-tolerant cells were involved in DNA conformation modification and chromosome organization. However, using topoisomerase inhibitors did not alter the killing dynamics of echinocandin drugs, suggesting that the pathways may not contribute to survival but instead serve other roles in tolerant cells. C. glabrata is known for its genome plasticity; its clinical isolates show remarkable genetic diversity both in terms of nucleotide sequence and chromosome structure (37–39). However, it is not known under what circumstances these genomic changes are acquired, as cells cultured in rich medium in the lab appear to be karyotypically stable (37). Thus, cells dealing with long-term echinocandin-induced stress may activate mechanisms enabling chromosomal variation to enhance adaptability. In this context, it is also interesting that we did not detect an increase in abundance of the “DNA damage histone” γH2A.X during echinocandin exposure, suggesting that DNA double-strand breaks (which induce γH2A.X formation) either may not be the main lesions triggering chromosomal alterations or that they occur at lower levels than can be detected by Western blotting. Nevertheless, the transcriptional upregulation of DNA topology and chromosomal conformation genes suggests that these cells may be in a specialized activated transcriptional state that resists drug stress and has an elevated genetic potential to evolve into a genotypically stable drug-resistant cell. Further studies addressing the development of resistant mutations in echinocandin-tolerant cells pre-exposed to this drug class will shed light on this issue.

Most transcriptional studies of fungal species exposed to drugs have been performed using short-term treatments and traditional bulk RNA-seq approaches with no enrichment for surviving cells (15, 16, 40). Because echinocandins rapidly induce cell death (17), such bulk analysis performed after a few hours of treatment overwhelmingly includes dead and dying cells. In contrast, our RNA-seq approach was designed to enrich for the rare C. glabrata cells surviving 24 hours of above-MIC drug exposure. Thus, our approach identified a distinct transcriptional profile of echinocandin-tolerant cells compared with other studies. Because the echinocandins target cell wall assembly, previous analyses of echinocandin-induced transcriptional changes mainly focused on cell wall-related processes. For instance, Xiong et al. described two C. albicans transcription factors regulating cell wall maintenance and homeostasis (Efg1 and Cas5) involved in the transcriptional response to 2-hour caspofungin treatment (15). Likewise, in Aspergillus fumigatus, it has been reported that caspofungin exposure leads to communication between the cell wall integrity and the high osmolarity-glycerol pathways through MAPK signaling (41). Other studies have also identified transcription factors that play a role in the regulation of the fungal cell wall as well as in echinocandin tolerance (40). Although our study also identified several upregulated cell wall maintenance genes in echinocandin-tolerant cells, including *FKS1* and *FKS2*, the cell wall integrity pathway as a whole was not present among the upregulated FUNCAT categories, suggesting that during long-term echinocandin exposure, C. glabrata cells may switch to other strategies to survive.

In most examined human-pathogenic yeasts and molds, amphotericin B and azoles trigger intracellular ROS formation, which contributes to the antifungal action of these drugs (24, 25, 42, 43). Echinocandins are also associated with an increase in ROS production (25, 43, 44), and at least in C. albicans, ROS scavenger thiourea caused a decrease in micafungin-induced killing (43). Also in C. albicans, genes involved in oxidative stress responses are induced in response to caspofungin (44). Our results (both RNA-seq and qRT-PCR of individual oxidative stress responder and regulator genes) show that although echinocandins also induce robust ROS formation in C. glabrata, this fungus does not respond to echinocandins by upregulating oxidative stress response pathways. Indeed, the high ROS levels might be a consequence of the downregulation of expression of the ROS detoxifying enzymes. Furthermore, we could not elicit a reduction of ROS using thiourea or several other scavengers, with the exception of ascorbic acid, which strongly reduced ROS levels in echinocandin-treated cells. Despite this decrease, however, ascorbic acid did not alter the dynamics of cell killing, indicating that in C. glabrata, the ROS do not significantly contribute to cell death but rather serve another function. Consistent with this idea, the ROS increase was not associated with detectable lipid peroxidation or DNA damage. Although these results run contrary to the traditional view that ROS are exclusively damaging and lack other physiological functions, they are consistent with observations that C. glabrata is exceptionally resistant to oxidative stress (45). In fact, it has recently been described that petite mutants of C. glabrata show a lack of oxidative stress susceptibility when incubated with hydrogen peroxide which is combined with a constitutive upregulation of environmental stress-response and heat shock protein-encoding genes (46). Relative to other species, C. glabrata exhibits a very restrained transcriptional response to phagocyte-induced ROS with insignificant upregulation of catalase (47). Interestingly, recent evidence suggests a critical role of ROS in the communication between the mitochondria and other cellular processes to maintain homeostasis and promote adaptation to stress (48). Furthermore, some studies have also proposed a role for oxidative stress adaptation in promoting virulence and drug resistance in C. glabrata, as several genes implicated in virulence, biofilm formation, and drug transport were also induced during the oxidative adaptation response (45). These studies, together with our results, suggest that in C. glabrata responding to echinocandins, ROS function as a signaling intermediate with as-yet-unknown specific downstream targets and contribute to fitness.

The involvement of mitochondria in echinocandin tolerance in C. glabrata was suggested by the RNA-seq data and the observation that mitochondrial inhibitors reduced C. glabrata killing by echinocandins over a range of concentrations. In contrast, however, deletion mutants with defective respiratory chain functions and deletions of several other mitochondrial genes, as well as a series of respiration-deficient petite strains of unknown etiology, were all hypotolerant to echinocandins, showing that mitochondrial inhibition and genetically encoded mitochondrial deficiency have opposite effects on echinocandin tolerance. Reduced tolerance to echinocandins in some mitochondrial mutants was previously described in C. albicans and S. cerevisiae. For instance, several C. albicans and S. cerevisiae mitochondrial mutants are sensitive to multiple cell wall-damaging agents, including calcofluor white, Congo red, and echinocandins, indicating a general cell wall defect (49, 50). This defect has been attributed to the role of mitochondria in phospholipid metabolism, whereby mitochondrial defects compromise phospholipid production, affecting cell wall biogenesis (49, 51). Similarly, S. cerevisiae mutants defective in producing cardiolipin, a predominantly mitochondrial lipid, have general cell wall defects (52). Interestingly, none of our C. glabrata mitochondrial mutants showed increased sensitivity to Congo red or calcofluor white, indicating that the mitochondrial effect on the cell wall in C. glabrata may be limited to β-glucans. Possibly relatedly, in S. cerevisiae, abrogation of the cardiolipin biosynthetic pathway results in reduced β-glucan levels and can be rescued by mutation of *KRE5*, which encodes a β-1,6-glucan synthase (52). A direct link between glucan synthesis and plasma membrane lipid composition has also been demonstrated in Aspergillus fumigatus, where two sphingolipids, namely, dihydrosphingosine and phytosphingosine, rendered GS insensitive to echinocandins (35). Thus, although our RNA-seq analysis did not reveal a lipid-related transcriptional signature in echinocandin-tolerant cells, the studies described above and our results obtained with mitochondrial mutants support a model in which mitochondrial status may influence the plasma membrane lipid composition, which in turn may influence echinocandin sensitivity.

Another potential link between mitochondria and echinocandin sensitivity has been suggested by reports indicating that in C. albicans and in C. glabrata echinocandins induce cell death by apoptosis (programmed cell death) and by necrosis (53, 54). It is well known that the role of mitochondria in programmed cell death is complex and that mitochondria have both pro- and antiapoptotic functions (55). Thus, it may not be surprising that inhibition of certain mitochondrial activities leads to different survival phenotypes than a total lack of certain mitochondrial proteins due to gene deletions. Interestingly, our results suggest that at least one of the mitochondrial inhibitors, rotenone, which elicited the strongest rescue of cell survival, may not be acting solely via the mitochondria. Rotenone is typically considered a mitochondrial complex I inhibitor. However, C. glabrata, like some other fungal species, has lost the canonical NADH:ubiquinone oxidoreductase (complex I) while retaining other mitochondrial oxidative phosphorylation components, including NADH dehydrogenases (encoded by *NDI1*, *NDE1*, and *NDE2*), which largely perform the same function as complex I. The observation that rotenone improved survival not only in wild-type cells but also in every examined mitochondrial mutant suggests that this rotenone effect is achieved not only via mitochondrial modulation but also via influencing other, as yet unclear secondary pathways. To identify these pathways, transcriptomic studies will likely be highly informative. In contrast, DPI (an inhibitor of NADH dehydrogenases) and sodium azide (an inhibitor of complex IV) both failed to improve survival in mitochondrial mutants, suggesting that their activity is indeed mediated by the mitochondria. Surprisingly, the transcriptome of C. glabrata echinocandin-tolerant cells showed a downregulation of multiple genes involved in mitochondrial function, e.g., *COX8*, *COX26*, *COA1*, *NDI1*, *QCR6*, *QCR8*, and *RCF1* (Data Set S1), which is the opposite to what has been described for A. fumigatus cells isolated during the caspofungin paradoxical effect (16). Thus, it seems that the mechanism responsible for echinocandin tolerance in C. glabrata and A. fumigatus may be different. This conclusion is also supported by the opposite effects of rotenone in the two systems (16).

In summary, our results reveal a thus far unappreciated complexity of the mitochondrial involvement in C. glabrata response to echinocandins and echinocandin-mediated killing. Although we show that the traditionally frequently used mitochondrial inhibitors reduce fungal killing by echinocandins, which is not a desired effect in the clinic, our results suggest that it may be possible to target fungal mitochondria to sensitize C. glabrata to these drugs. Indeed, other types of mitochondrial inhibitors, e.g., those that interfere with mitochondrial biogenesis by blocking mitochondrial protein import (56), may be investigated in future studies as potential enhancers of echinocandin fungicidal action.

## MATERIALS AND METHODS

### Yeast strains and media.

The C. glabrata strains used in this study were ATCC 2001 and mutants (gene deletions and petite mutants) derived from ATCC 2001 (Table S3). Cells were cultured in standard yeast extract-peptone-dextrose (YPD) medium at 37°C.

### RNA sequencing for echinocandin enriched tolerant cells.

Overnight cultures containing 10^7^/ml C. glabrata cells were exposed to 0.25 μg/ml of caspofungin or 0.06 μg/ml of micafungin. After a 24-hour incubation at 37°C with shaking (150 rpm), cells were then collected by filtration using 0.45-μm mixed cellulose ester membrane filters (Millipore) and resuspended in phosphate-buffered saline (PBS) stained with 10 μg/ml propidium iodide (PI) and sorted using the FACSMelody system (BD Biosciences). Logarithmically growing no-drug controls were collected after 2 hours of growth in drug-free YPD, whereas stationary-phase controls were collected after 24 hours of growth in drug-free YPD. Each sample was collected by FACS, treated with zymolyase (5 U per 10 μl of cells) for 30 minutes at 37°C to digest the cell walls, and then pipetted into a 96-well plate to produce ∼10 PI-negative cells per well. Thirty-six biological replicates were submitted for each echinocandin-treated sample (caspofungin and micafungin), and 12 biological replicates were submitted for each control (logarithmic and stationary). The samples were sent to Columbia University Medical Center (New York, NY) for library preparation, and the enriched RNA sequencing of ∼10 live cells per well was performed as described previously (57) with some modifications for the reverse-transcription reaction using 40 U Maxima H (ThermoFisher), 2× Maxima H buffer (ThermoFisher), 4 U SuperaseIN (ThermoFisher), 15% polyethylene glycol, and 2 μM template switching oligonucleotide (IDT) in a total volume of 7.5 μl.

For the RNA-seq analysis, reads were aligned to the NCBI reference GCF_000002545.3_ASM254v2, using STAR (58). Alignments were assigned to genes using featureCounts (59). The bam files produced by featureCounts were sorted and indexed with SAMtools (60). The package umi_tools was used to assemble a count matrix (61). Wells were filtered by removing outliers in the distributions of total counts, genes detected, and mitochondrial expression. Highly abundant ribosomal RNAs, which would interfere with normalization, were removed prior to normalization and differential expression analysis. The package sctransform was used to normalize count data (62), and Seurat was then used to perform differential expression testing with the Wilcoxon signed-rank test between groups (63). Raw RNA-seq data have been deposited at the Gene Expression Omnibus (accession no. GSE178797), and differential expression gene (DEG) files are shown in Data Set 1. The list of C. glabrata*-*S. cerevisiae direct orthologs was downloaded from http://www.candidagenome.org/download/homology/orthologs and supplemented by manual curation of C. glabrata genes using http://www.candidagenome.org. Functional enrichment analysis (FUNCAT) was performed using FungiFun2 (https://elbe.hki-jena.de/fungifun/) (64). The heatmap was generated using R studio.

### RNA isolation and bulk transcriptome analysis.

Cultures containing 10^7^/ml C. glabrata were exposed to 0.06 μg/ml of micafungin for 24 h and collected in triplicate in the same fashion as explained in the previous section. Stationary-phase cells were collected after 24 hours of growth in drug-free YPD in duplicate as a no-drug control. Total RNA was extracted using the RNeasy minikit (Qiagen Science) following the manufacturer’s instructions. The RNA was then treated with RNase-free DNase (ThermoFisher) according to the manufacturer’s recommendations and stored at −80°C until RNA sequencing was performed by Genewiz (South Plainfield, NJ). The RNA-seq data were analyzed using software (Basepair, New York, NY) with a pipeline as follows: reads were aligned to the transcriptome derived from sacCer3 using STAR with default parameters, read counts for each transcript were measured using featureCounts and the differentially expressed genes were determined using DESeq2, and a cutoff of 0.05 for the adjusted *P* value (corrected for multiple hypotheses testing) was used for creating differentially expressed gene lists (Data Set 1). Gene set enrichment analysis (GSEA) was performed on normalized gene expression counts, using gene permutations for calculating the *P* value. The fastq RNA-seq files have been deposited at the Gene Expression Omnibus (accession no. GSE178656).

### Antifungal susceptibility testing.

Micafungin (Astellas), caspofungin (Merck), and fluconazole (LKT Labs) susceptibility testing was performed using a broth microdilution method following CLSI standards (65) with some modifications. The medium used was YPD broth, and the concentrations tested ranged from 0.0035 to 2 μg/ml to echinocandins and 0.25 to 128 μg/ml to fluconazole. MICs were visually read after 24 hours of incubation at 37°C. At least three biological replicates were performed.

### Cell survival measurements after caspofungin treatment combined with inhibitors.

To measure the effects of topoisomerase or mitochondrial inhibitors on cell survival in the presence of caspofungin, overnight cultures containing 10^7^ cells/ml were resuspended in fresh YPD and added to caspofungin plus one of the inhibitors at the following concentrations. The selected concentrations for the topoisomerase inhibitors were 3 μM, 200 μM, and 100 μM doxorubicin (ThermoFisher), etoposide (Millipore Sigma), and topotecan (Cayman Chemical), respectively; all inhibitors were dissolved in dimethyl sulfoxide (DMSO). The selected concentrations for the mitochondrial inhibitors or ROS scavengers were 0.3 mM, 0.005 mM, 0.5 mM, and 50 mM rotenone, diphenyleneiodonium chloride, sodium azide, and ascorbic acid (Millipore Sigma), respectively; rotenone and diphenyleneiodonium chloride were dissolved in DMSO, whereas sodium azide and ascorbic acid were dissolved in water. The selected concentration of rotenone was determined by its solubility in liquid YPD because concentrations higher than 0.3 mM resulted in precipitation. After 24 hours of drug exposure, aliquots were harvested and dilutions plated on drug-free YPD plates. The percentage of survival was calculated based on the observed number of colonies from caspofungin cultures relative to the corresponding colony counts from non-caspofungin treated cultures. At least three biological replicates were performed for every strain and condition.

### Reactive oxygen species quantification.

The presence of intracellular reactive oxygen species (ROS), more specifically hydrogen peroxide, was assessed by the cell-permeant 2′,7′-dichlorodihydrofluorescein diacetate (CFDA) (10 μg/ml; ThermoFisher) according to the manufacturer’s instructions. Cells were collected and washed with PBS, and then 10 minutes before flow cytometric analysis, each sample was also stained with 10 μg/ml PI to exclude PI-positive dead cells. The superoxide indicator dihydroethidium (DHE) was used to assess superoxide radical species (6.5 μg/ml in DMSO; ThermoFisher) following the manufacturer’s instructions. In this case, dead cells to be excluded from the analysis were stained with Sytox green (1 μM; ThermoFisher). Percentages of ROS-positive cells were calculated relative to all live (PI or Sytox green negative) cells after incubation with the dyes. Data were analyzed using FlowJo software v10.6.1 (BD Biosciences).

### RNA extraction and quantitative real-time reverse-transcription PCR (qRT-PCR).

Overnight cultures containing 10^7^ C. glabrata cells/ml were resuspended in fresh YPD containing echinocandins (0.25 μg/ml of caspofungin or 0.06 μg/ml of micafungin); harvested after 2, 6, or 24 hours; filtered; and concentrated in the same fashion as for the enriched RNA-seq transcriptomic analysis, except that cells were resuspended in RNA extraction buffer provided by the Qiagen kit instead of PBS. Nontreated controls (logarithmic- and stationary-phase cells) and H_2_O_2_-treated controls (50 mM) were harvested too in the same fashion, whereby the volume of overnight culture needed to obtain a big enough cell pellet to isolate RNA was smaller. Total RNA was extracted using the RNeasy minikit (Qiagen Science) following the manufacturer’s instructions. The RNA was then treated with RNase-free DNase (ThermoFisher) according to the manufacturer’s recommendations. RNA samples were stored at −80°C. The transcript levels of *SOD2*, *CTA1*, *GRX2*, *YAP1*, *MSN2*, and *MSN4* were measured by RT-PCR using one-step SYBR PrimeScript RT-PCR kit II (TaKaRa). Reactions were run on an Mx3005P qPCR System (Agilent Technologies) containing 10 ng of the RNA sample, 0.4 μM of each primer (Table S4), 12.5 μl of 2 ×  one-step SYBR RT-PCR buffer, and 1 μl of PrimeScript 1 step enzyme mix 2 in a volume of 25 μl. Thermal cycling conditions were 42°C for 5 min for the reverse transcription and PCR cycling with initial denaturation at 95°C for 10 s; followed by 40 cycles of denaturation at 95°C for 5 s and annealing and elongation at 60°C for 20 s; a post-PCR melting curve analysis with 95°C for 5 s; 60°C for 1 min; and then increasing to 95°C.

10.1128/mBio.01959-21.9 TABLE S4 Sequences of primers used in this study for qRT-PCR transcriptional experiments and generation of CRISPR knockout mutants. *Primers described in a different study (Q. Q. Li, J. Skinner, and J. E. Bennett, BMC Mol Biol 13:22, 2012, doi:https://doi.org/10.1186/1471-2199-13-22). Download Table S4, DOCX file, 0.02 MB. Copyright © 2021 Garcia-Rubio et al. 2021 Garcia-Rubio et al. https://creativecommons.org/licenses/by/4.0/ This content is distributed under the terms of the Creative Commons Attribution 4.0 International license.

Each experiment was carried out in biological triplicates, and at least two technical triplicates of each biological replicate were also performed. Negative controls were included in each run. The *RDN5.8* gene was used as the reference gene to normalize the data (66). Comparative expression analyses were performed using the threshold cycle (2^−ΔΔCT^ method) (67). The fold changes were determined from the normalized expression of the average of treated samples relative to the normalized expression of the average of stationary nontreated controls. Calculations were also done using nontreated logarithmic-phase controls; all trends were similar, so these data were not shown.

### *In vitro* echinocandin tolerance assay.

Overnight cultures containing 10^7^ C. glabrata cells were resuspended in fresh 1 ml YPD and incubated at 37°C with shaking (150 rpm) for 24 hours in the presence of a range of caspofungin concentrations (0, 0.016, 0.06, 0.25, 1, and 4 μg/ml). After 24 hours, 0.1 ml of the appropriate dilutions for each culture was plated onto YPD plates. CFUs were determined, and survival percentage was obtained by normalizing the CFU obtained from cultures treated with the indicated concentration of drug to nontreated controls. Cotreatment with mitochondrial inhibitors or ROS scavengers was performed in the same way as described above, except when there was overnight pre-exposure to the mitochondrial inhibitors before caspofungin was added to the media. At least three biological replicates were performed for every strain and condition.

### Lipid peroxidation.

Suspensions of 10^7^ C. glabrata stationary-phase cells/ml were exposed to caspofungin (0.25 and 1 μg/ml) or hydrogen peroxide (5 and 10 mM) and incubated with or without 50 μM diphenyl-1-pyrenylphosphine (DPPP) (ThermoFisher) at 37°C (68, 69). The fluorescence intensity was measured every hour using a Tecan infinite 200 Pro plate reader with a 340/380 nm excitation filter. Data are shown in arbitrary units of fluorescence intensity after 2 and 6 hours of caspofungin exposure.

### Western blotting.

C. glabrata cells exposed to echinocandin drugs (0.25 μg/ml of caspofungin or 0.06 μg/ml of micafungin), as well as positive (50 mM H_2_O_2_) and untreated (stationary or logarithmic-phase cells) control samples, were collected after 2, 6, and 24 hours of growth, except for H_2_O_2_ where 24-hour cultures had too few cells to proceed. Whole-cell lysates were prepared by trichloroacetic acid (TCA) precipitation. Briefly, cell pellets were resuspended in 20% TCA, broken by bead beating, and washed twice with 5% trichloroacetic acid (TCA). Proteins were pelleted, resuspended in sodium dodecyl sulfate-polyacrylamide gel electrophoresis (SDS-PAGE) loading buffer, incubated at 95°C for 5 min, and centrifuged prior to loading on 16% acrylamide gels. Antibodies were obtained commercially, namely, anti-H2A (catalog no. 39945; Active Motif) and anti-γH2A.X (ab15083; Abcam).

### Spotting assay.

Cells obtained from overnight cultures grown in YPD broth at 37°C were spotted in serial 10-fold dilutions on YP plates containing 2% glycerol or YPD plates containing 0.05 mg/ml of Congo red (Millipore Sigma) or 0.01 mg/ml of calcofluor white (Millipore Sigma). The growth of each strain was evaluated after 24 hours of incubation at 37°C. Petite mutants grew much slower than the other mutants, so their growth was also evaluated after 48 hours.

### Microscopy mitochondrial detection.

Mitochondria were stained with the fluorescent dye MitoTracker Green FM (ThermoFisher) according to the manufacturer’s protocol but with some modifications for yeast staining. The cells were harvested by centrifugation, diluted to 10^6^ cells/ml in 10 mM HEPES buffer (pH 7.4) containing 5% of glucose, and stained for 30 min with 0.1 μM MitoTracker Green at room temperature. Stained cells were visualized using a Nikon Eclipse Ti2 inverted microscope (488-nm filter) with a Hamamatsu ORCA-Flash4.0 camera and analyzed using NIS-Elements software.

### Construction of C. glabrata deletion mutants.

Deletion mutants were generated in house using a CRISPR-Cas9-targeted integration replacing the desired open reading frame (ORF) by a nourseothricin (NAT) resistance cassette (Table S3). The deletion constructs were generated by PCR using ∼100-nucleotide-long primers designed to amplify NAT and also containing homology regions flanking the locus of interest. All primers used are listed in Table S4. Gene replacement by the deletion cassettes was performed using CRISPR as described previously (70). Briefly, cells were made competent for electroporation using the Frozen-EZ yeast transformation kit (Zymo Research) according to the manufacturer’s instructions and then electroporated with a Cas9-genomic RNA (gRNA) complex (Integrated DNA Technologies, Coralville, IA) and the DNA containing the deletion construct. Transformants were selected on NAT-containing plates and validated by PCR amplification and sequencing of the targeted locus using external primers (Table S4). At least two independent transformants were generated and analyzed for every deletion mutant. All primers were ordered from Integrated DNA Technologies, and all Sanger sequencing of the above-described constructs was done by Genewiz.

### Measurement of inhibition of glucan synthase by micafungin.

Three isogenic ATCC 2001 background strains were selected to examine glucan synthase (GS) inhibition by echinocandins *in vitro*, namely, wild-type, *ndi1Δ*, and caspofungin-derived petite mutant 1. The strains were grown with vigorous shaking at 37°C to early stationary phase in YPD broth, and cells were collected by centrifugation. Cell disruption, membrane protein extraction, and partial GS purification by-product entrapment were performed as described previously (36). Sensitivity to micafungin was measured at least in triplicate in a polymerization assay using a 96-well multiscreen high-throughput screen filtration system (Millipore) with a final volume of 100 μl. Serial dilutions of micafungin (0.01 to 10,000 ng/ml) were used to determine 50% inhibitory concentration (IC_50_) values. Inhibition profiles and IC_50_ values were determined using a sigmoidal response (variable-slope) curve-fitting algorithm with GraphPad Prism 8 software.

### Statistical analysis.

All shown results represent an average of three or more independent experiments (biological replicates). Error bars represent the standard deviation. All data were analyzed using GraphPad Prism 8 software. Statistical analyses were performed using an unpaired *t* test to determine significant differences between experimental groups. *P* values of ≤0.05 were considered statistically significant.
