# Supplementary material for: Multifactorial Role of Mitochondria in Echinocandin Tolerance Revealed by Transcriptome Analysis of Drug-Tolerant Cells
Source: mBio. 2021 Aug 10;12(4):e01959-21. doi: 10.1128/mBio.01959-21 (PMC8406274; doi:10.1128/mBio.01959-21)
Supplement: TABLE S1 [file mbio.01959-21-st001.docx]

|  | **CSF** | | **MCF** | |
| --- | --- | --- | --- | --- |
|  | **Average (%)** | **SD** | **Average (%)** | **SD** |
| **No-dye** | 0.0003 | 0.0005 | 0.0003 | 0.0000 |
| **PI** | 0.0528 | 0.0168 | 0.0413 | 0.0042 |
| **CFDA-AM** | 0.0000 | 0.0000 | 0.0010 | 0.0000 |
| **PI+CFDA-AM** | 0.0483 | 0.0071 | 0.0245 | 0.0318 |
| **SG** | 0.0020 | 0.0000 | 0.0210 | 0.0000 |
| **RFP+SG** | 0.0055 | 0.0064 | 0.0010 | 0.0000 |
| **GFP+PI** | 0.0100 | 0.0090 | 0.0067 | 0.0038 |
| **FUN-1** | 0.0005 | 0.0007 | 0.0003 | 0.0006 |
