# Supplementary figures and images for: Multifactorial Role of Mitochondria in Echinocandin Tolerance Revealed by Transcriptome Analysis of Drug-Tolerant Cells
Source: mBio. 2021 Aug 10;12(4):e01959-21. doi: 10.1128/mBio.01959-21 (PMC8406274; doi:10.1128/mBio.01959-21)

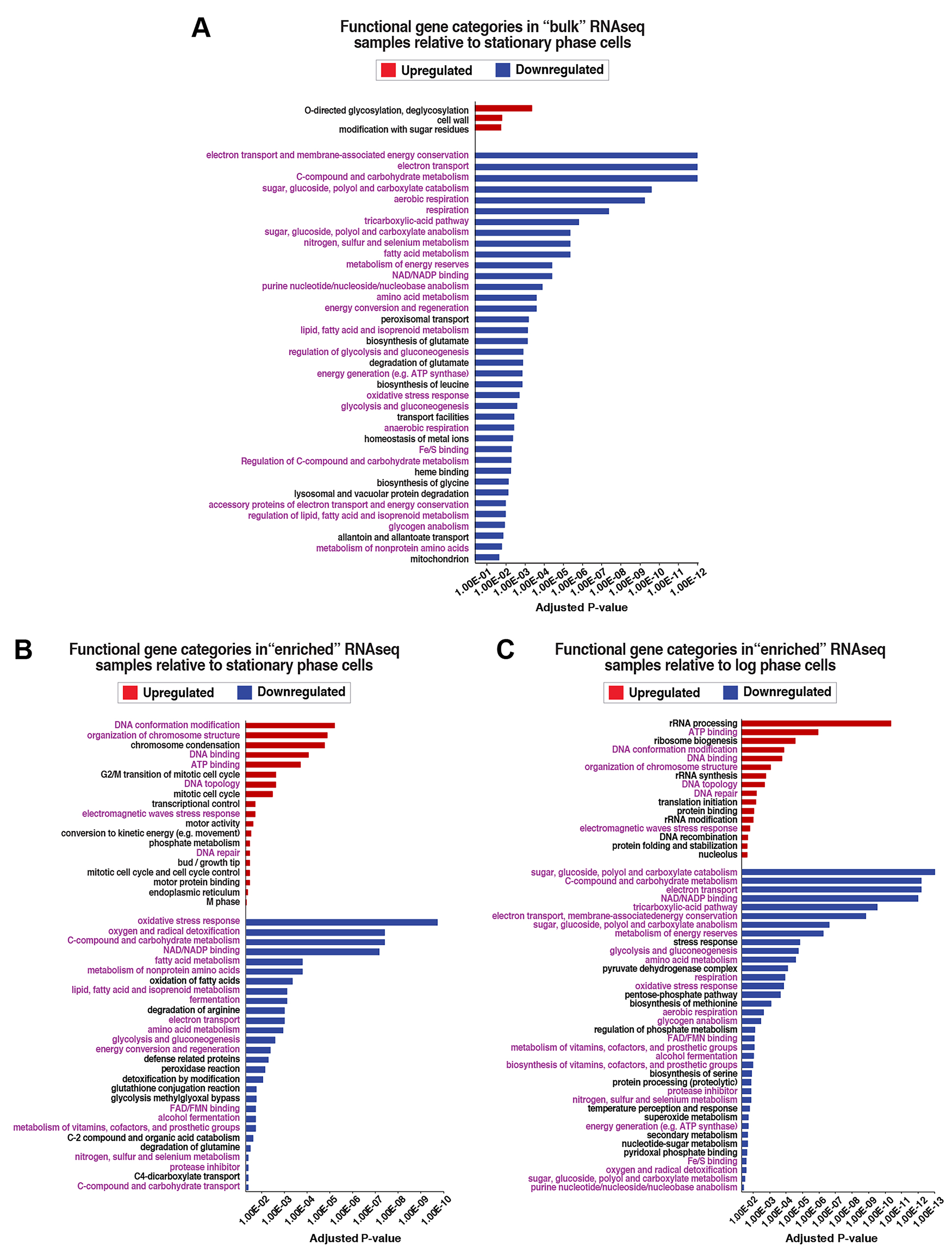

Supplement: FIG S1 [file mbio.01959-21-sf001.tif]

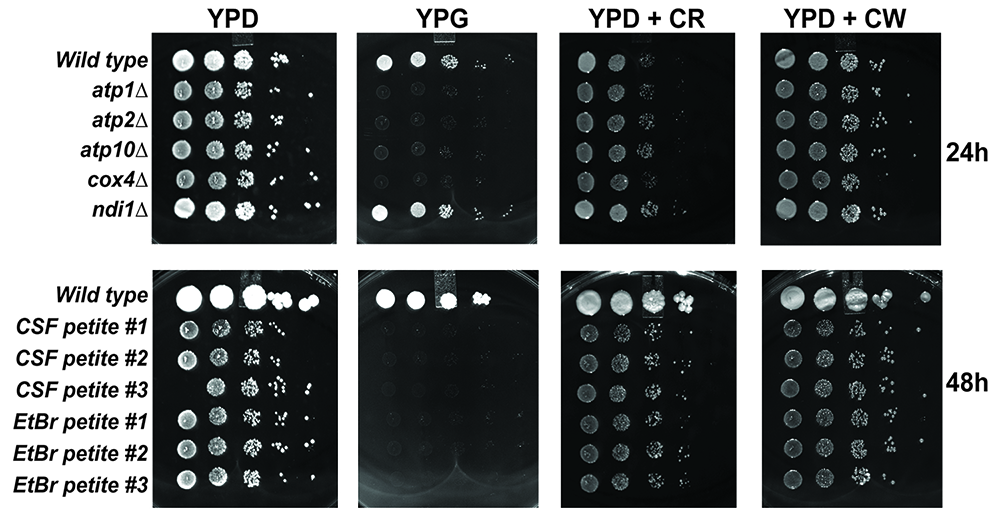

Supplement: FIG S2 [file mbio.01959-21-sf002.tif]

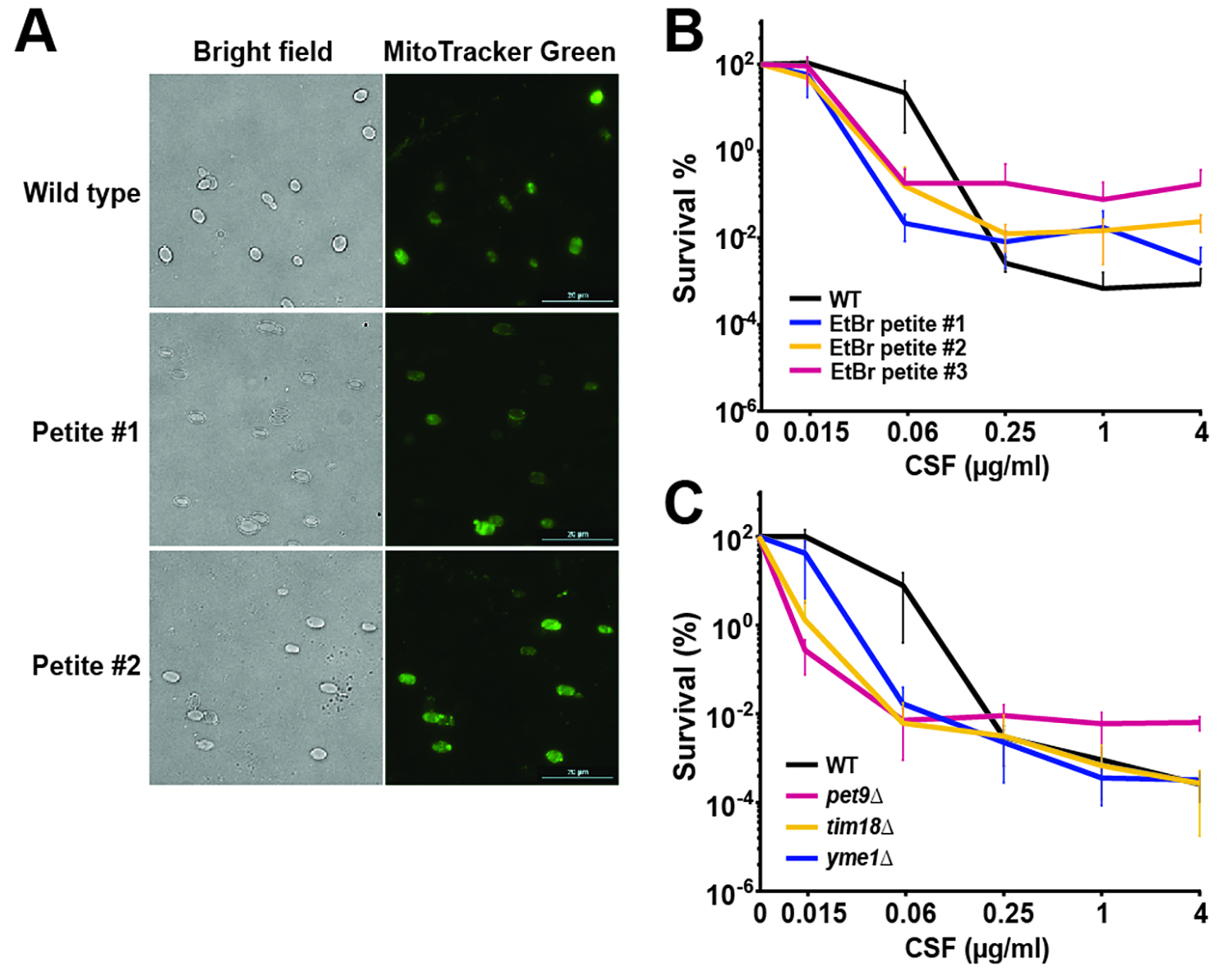

Supplement: FIG S3 [file mbio.01959-21-sf003.tif]

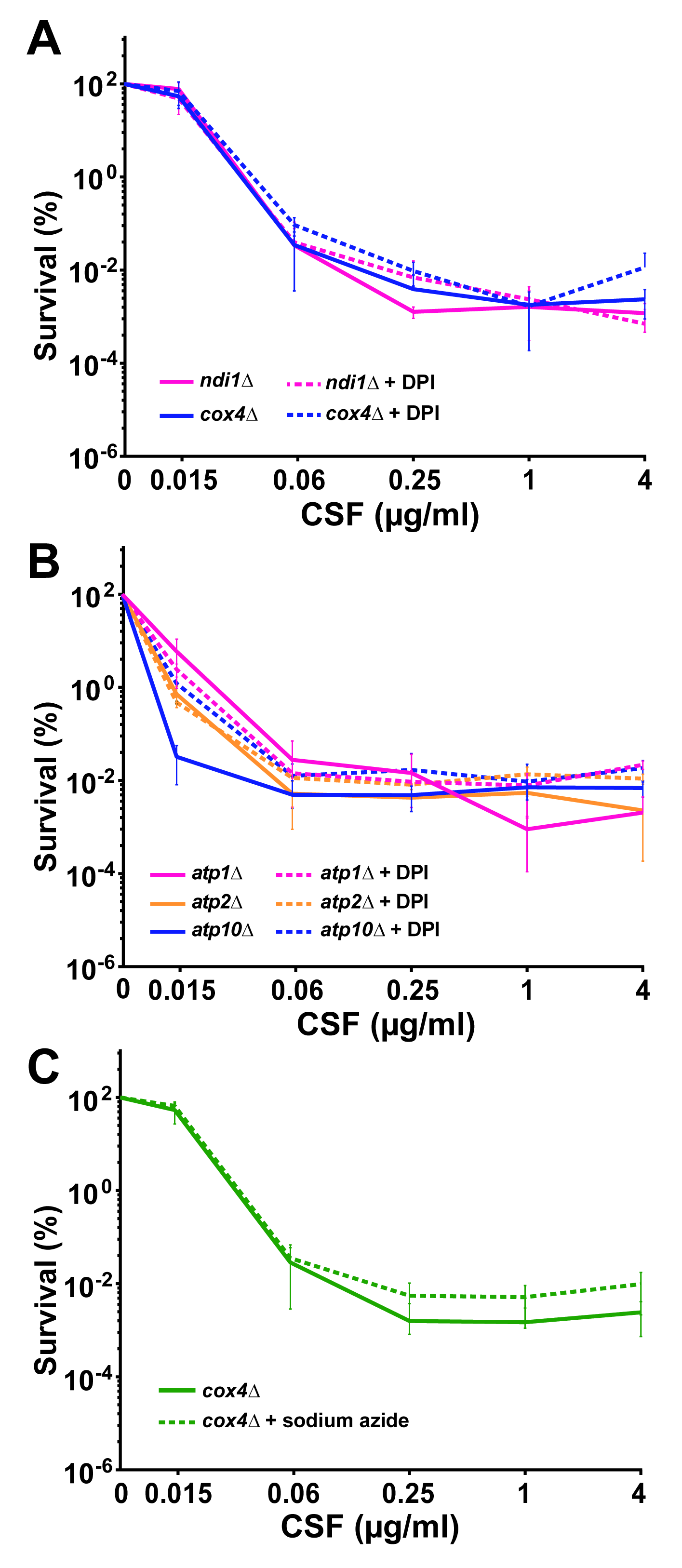

Supplement: FIG S4 [file mbio.01959-21-sf004.tif]

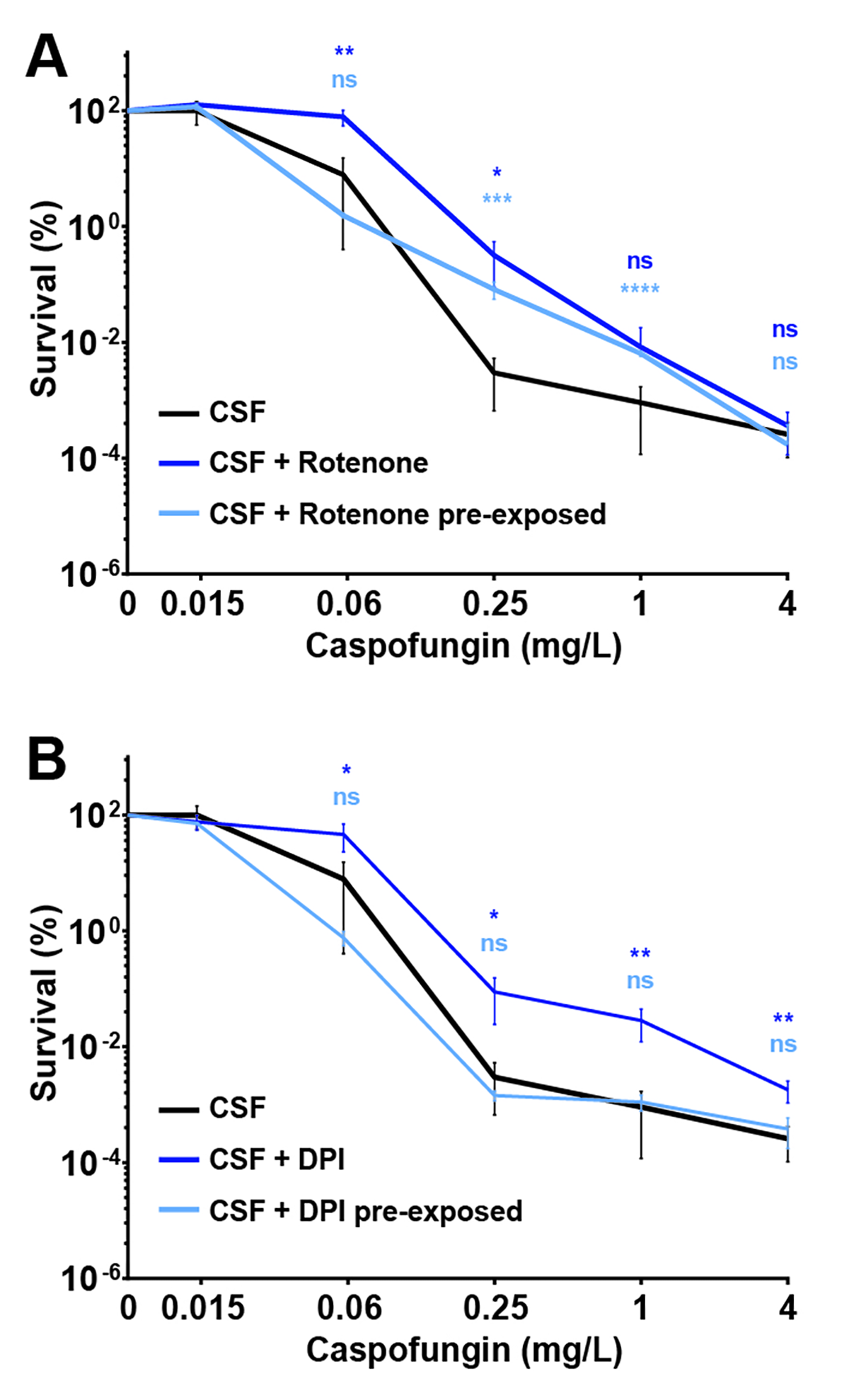

Supplement: FIG S5 [file mbio.01959-21-sf005.tif]
