# Supplementary material for: Multifactorial Role of Mitochondria in Echinocandin Tolerance Revealed by Transcriptome Analysis of Drug-Tolerant Cells
Source: mBio. 2021 Aug 10;12(4):e01959-21. doi: 10.1128/mBio.01959-21 (PMC8406274; doi:10.1128/mBio.01959-21)
Supplement: TABLE S2 [file mbio.01959-21-st002.docx]

|  |  | |  |  | | |  | |  |
| --- | --- | --- | --- | --- | --- | --- | --- | --- | --- |
| **Inhibitor alone** | | **MIC (mM)** | **Selected concentration (mM)** | | **Inhibitor**  **+ echinocandin** | **CSF MIC (µg/ml)** | | **MCF MIC (µg/ml)** | |
|  | |  |  | | No inhibitor | 0.12 | | 0.03 | |
| Topotecan | | >0.2 | 0.1 | | Topotecan | 0.12 | | 0.03 | |
| Etoposide | | 0.625 | 0.2 | | Etoposide | 0.12 | | 0.03 | |
| Doxorubicin | | 0.0125 | 0.003 | | Doxorubicin | 0.12 | | 0.03 | |
| Rotenone | | >0.3^*^ | 0.3 | | Rotenone | 0.12 | | 0.03 | |
| DPI | | 0.015 | 0.005 | | DPI | 0.12 | | 0.03 | |
| Sodium azide | | 1.5 | 0.5 | | Sodium azide | 0.12 | | 0.03 | |
| Ascorbic acid^**^ | | >50 | 50 | | Ascorbic acid | 0.12 | | 0.03 | |
