# Supplementary material for: Multifactorial Role of Mitochondria in Echinocandin Tolerance Revealed by Transcriptome Analysis of Drug-Tolerant Cells
Source: mBio. 2021 Aug 10;12(4):e01959-21. doi: 10.1128/mBio.01959-21 (PMC8406274; doi:10.1128/mBio.01959-21)
Supplement: TABLE S3 [file mbio.01959-21-st003.docx]

| **Name** | **Strain description** | **Systematic gene name** | **Fluconazole**  **MIC (µg/ml)** |
| --- | --- | --- | --- |
| ATCC2001 | Wild-type parental strain | - | 64 |
| *ndi1∆* | CRISPR-generated *ndi1* knock-out with pTEF1-NAT | CAGL0B02431g | 64 |
| *cox4∆* | CRISPR-generated *cox4* knock-out with pTEF1-NAT | CAGL0L06160g | 64 |
| *atp1∆* | CRISPR-generated *atp1* knock-out with pTEF1-NAT | CAGL0M09581g | 128 |
| *atp2∆* | CRISPR-generated *atp2* knock-out with pTEF1-NAT | CAGL0H00506g | 128 |
| *atp10∆* | CRISPR-generated *atp10* knock-out with pTEF1-NAT | CAGL0C02651g | 128 |
| *pet9∆* | CRISPR-generated *pet9* knock-out with pTEF1-NAT | CAGL0F04213g | 32 |
| *tim18∆* | CRISPR-generated *tim18* knock-out with pTEF1-NAT | CAGL0A03784g | 64 |
| *yme1∆* | CRISPR-generated *yme1* knock-out with pTEF1-NAT | CAGL0K05093g | 64 |
| EtBr #1 | Ethidium bromide-derived petite mutant | - | >128 |
| EtBr #2 | Ethidium bromide-derived petite mutant | - | >128 |
| EtBr #3 | Ethidium bromide-derived petite mutant | - | >128 |
| ECH #1 | Caspofungin-derived petite mutant | - | >128 |
| ECH #2 | Caspofungin-derived petite mutant | - | >128 |
| ECH #3 | Caspofungin-derived petite mutant | - | >128 |
