# Supplementary material for: Multifactorial Role of Mitochondria in Echinocandin Tolerance Revealed by Transcriptome Analysis of Drug-Tolerant Cells
Source: mBio. 2021 Aug 10;12(4):e01959-21. doi: 10.1128/mBio.01959-21 (PMC8406274; doi:10.1128/mBio.01959-21)
Supplement: TABLE S4 [file mbio.01959-21-st004.docx]

| **Gene** | **Description** | **Sequence (5’-3’)** |
| --- | --- | --- |
| *SOD2* | RTqPCR | AGCTGCACGTGATCTAATCC |
|  | RTqPCR | CTTCACCACCACCGTTCTT |
| *CTA1* | RTqPCR | GAAGGGTGAATGGCACTATGT |
|  | RTqPCR | CTGGGTTCTTACCAGCGATTT |
| *GRX2* | RTqPCR | GCAAACCCTGTTCGAAGAATTG |
|  | RTqPCR | CAGCCAAAGCTTGTTGAATGT |
| *YAP1* | RTqPCR | AACTCGGTTGCCGCTAAT |
|  | RTqPCR | ATGAGGTTGGGTCTGTGTTC |
| *MSN2* | RTqPCR | CGACTGGCAAGGACAAGAA |
|  | RTqPCR | AAGGCAACGCACCATTAGA |
| *MSN4* | RTqPCR | CACTGACTTCGCCAACTAGAA |
|  | RTqPCR | AGGACAGCATCTTCCCAAAG |
| *RDN5.8^*^* | RTqPCR housekeeping gene | CTTGGTTCTCGCATCGATGA |
|  | RTqPCR housekeeping gene | GGCGCAATGTGCGTTCA |
| *ATP1* | fwd replacement template | AGGTTGGTCTGTTTTCCTGTGACTTGCAGTATACATTATTCAGTATACATCAAACCAAAAGAAAATAAAGTTTAAAAATAGCTTGCCTCGTCCCCGCCGG |
|  | rv replacement template | TTTGTGTGCGAAAAGAAAACAAACAAAATAGGAACAAAGCCTGTGCTGGTCACTGGGCGATGTGCTTTCAGAAAACCATCTGGATGGCGGCGTTAGTATC |
|  | fwd NAT validation | TACACGTGATTTCCCTGCCGAC |
|  | rv NAT validation | GGTAATAAACAAGGTTGTGTGCGG |
| *ATP2* | fwd replacement template | GATACATAGATACATAATAAGCTAAAAATTGTTTTTTTTTTTCAGACTTGTAACAACAACTCTAAGCTCAACTATTAACAGCTTGCCTCGTCCCCGCCGG |
|  | rv replacement template | CAAAATGTACAATAGTCGATTAAAACAAGGGAAAAAAAGTCGAGAGTATAAGTCTCGATTTTCATTCTGTTTTACCAACCTGGATGGCGGCGTTAGTATC |
|  | fwd NAT validation | CTCTCTGCCATACAAACTGCG |
|  | rv NAT validation | GCGCAGAGGGGAAAATGTCG |
| *ATP10* | fwd replacement template | TTAAGTGGCTCTGGTCATAAGTACACGATTGATCAACAACTAATAGCATAAAGCCTGAACCAAACTACTTTGAGTTAAATGCTTGCCTCGTCCCCGCCGG |
|  | rv replacement template | TGGATGCGGGCTAAAGTGGTGAATATATATTTTGCATTATGTCGTATATACATGCGATTTTGATCTATCTACAGAGTGGCTGGATGGCGGCGTTAGTATC |
|  | fwd NAT validation | CGGATCGGTAACTATCAATCACTG |
|  | rv NAT validation | CGTTTGGGACCGTTAGGGACC |
| *COX4* | fwd replacement template | GCTAACTTGAACCAACAATTTAGAATTAATAAAACCCAGTACTTTAACAACTACAAGACATAGATTAACAGAAGATAAAGGCTTGCCTCGTCCCCGCCGG |
|  | rv replacement template | CTTAGACATGAATAATAATATATAAATTTTCTCTTACTGGTGGCTCACCATCTAGTTCTTCTTTTCATGGGGTGTGGATCTGGATGGCGGCGTTAGTATC |
|  | fwd NAT validation | GATTTTCAAGAACAGAAGAAGCTACC |
|  | rv NAT validation | CTGTGTAAGTCTATCACTGCTGAG |
| *NDI1* | fwd replacement template | ACCACCAGCGTAGTATATACAAAACTATACAGCCGATAGGTGGAGTATAATAATAACAAGGAAACGAAAAACACCAAACCGCTTGCCTCGTCCCCGCCGG |
|  | rv replacement template | TGTGACACGTAGTTCTTACTACAAATAAATATGGGATGGCTCAATAAGGACGGGGGGGGAGGAGAAAACCGCAATGTATCTGGATGGCGGCGTTAGTATC |
|  | fwd NAT validation | CGAGACACATACGATACCCATCG |
|  | rv NAT validation | GGTGCTGAACGACAACATATACG |
| *PET9* | fwd replacement template | AAGGACTTCTTTTCGAACTTACTTCAAAGAAAAGAAAATAAAAATACTAATATAATATTCCTACACATACATATAACAGAGCTTGCCTCGTCCCCGCCGG |
|  | rv replacement template | CATAAACTGTCTAGTTGTTGTTGTTTTCCTGGATGCTGTTGAATTTTATTGATTATTGGTAGACGCTAGGTTAGTAAGACTGGATGGCGGCGTTAGTATC |
|  | fwd NAT validation | GGAAACTCGTGAAAGCTTGTGC |
|  | rv NAT validation | GTTAAACCCGATCCAATTCCGGG |
| *TIM18* | fwd replacement template | ACTGTCTGTTACACGATCGTCTAGGAGATCGTGACTTTTATTCTGGAAGAAATATAATACACAACAAGAGAGAAAGAACTGCTTGCCTCGTCCCCGCCGG |
|  | rv replacement template | TGCAATTTTGAAATAAGCTACATAAAGCAGTTTATATTATCTACATGGTTGAAATGATGCTAGTATTGATTTACTTTATCTGGATGGCGGCGTTAGTATC |
|  | fwd NAT validation | AGAATTTAACCGCCGATCTGGC |
|  | rv NAT validation | TGTATGCCTGATCCTAATGATTGC |
| *YME1* | fwd replacement template | ACTACTGCGCTATTGACCTCTTTGTTCTAATTAAAATTTCACGCGGGGGGTTAGGTACACATTTAGGTACAATATAAGAGGCTTGCCTCGTCCCCGCCGG |
|  | rv replacement template | GGTTAAAAAAAGTGGATCCATAAATGTCTATAATATACAATTCTCAAGTTTTACTTGATAAATCAACATCAATAGTATTCTGGATGGCGGCGTTAGTATC |
|  | fwd NAT validation | GCGATGTCCTGAACAACCGTAC |
|  | rv NAT validation | GCCGCCCCAGTGATCCAAAC |
| *NAT* | rv internal primer | GTATTCTGGGCCTCCATGTC |
|  | fwd internal primer | GTGAATGCTGGTCGCTATAC |
